# Passive Immunization Hesitancy Among Rabies-Exposed Individuals in China: A Multicenter Cross-Sectional Study

**DOI:** 10.3390/tropicalmed11090238

**Published:** 2026-08-24

**Authors:** Qisheng Hou, Xiaoliang Xu, Si Liu, Chen Sun, Fengfeng Zhu, Yang Yang, Jianchao Shao, Yifan Wang, Yanqiang Feng, Yingxiang Liu, Yan Yan, Song Wang, Yan Wang, Cheng Liu

**Affiliations:** 1 Department of Emergency, Peking University First Hospital, Beijing 100034, China; 2 Wuhan Jinyintan Hospital, Wuhan 430023, China; 3 Suzhou Fifth People’s Hospital, Suzhou 215000, China; 4 Department of Second Affiliated Hospital of Shantou University Medical College, Shantou 515041, China; 5 Shapingba District Center for Disease Control and Prevention, Chongqing 400000, China; 6 Pingdu People’s Hospital, Pingdu 266700, China; 7 The Fourth People’s Hospital of Datong City, Datong 037008, China; 8 Shenzhou Hospital of Integrated Traditional Chinese and Western Medicine, Shenzhen 518104, China; 9 Zhoukou Center for Disease Control and Prevention, Zhoukou 466000, China; 10 Nanxishan Hospital of Guangxi Zhuang Autonomous Region, Guilin 541002, China; 11 Shencheng Township Health Center of Yingshang County, Yingshang 236200, China

**Keywords:** rabies, post-exposure prophylaxis, passive immunization, treatment hesitancy

## Abstract

Background: Rabies is an almost universally fatal disease for which post-exposure prophylaxis (PEP), including passive immunization (PI) with rabies immunoglobulin or monoclonal antibodies, remains the only effective intervention. Despite established clinical guidelines, real-world PI utilization remains suboptimal. This study aimed to investigate the prevalence and determinants of PI hesitancy among previously unvaccinated patients with Category III rabies exposure in China. Methods: This multicenter cross-sectional questionnaire study was conducted at 12 rabies PEP clinics or emergency departments in 12 provinces of mainland China. The analysis included 585 previously unvaccinated adults with Category III rabies exposure who initially hesitated to receive or declined PI. The questionnaire collected sociodemographic characteristics, exposure profiles, awareness of the early protection gap, reasons for initial PI hesitancy or refusal, the decision to receive PI during the same clinical encounter, and PI product selection. Multivariable logistic regression identified factors associated with PI treatment decisions, with results reported as adjusted odds ratios (aORs); reason-specific models used the Benjamini–Hochberg false discovery rate correction. All inferential analyses were restricted to adults. Results: Among 5068 eligible previously unvaccinated patients with Category III rabies exposure, 2866 (57%) initially hesitated to receive or declined recommended PI. Of 617 questionnaire respondents, 32 minors were excluded, leaving 585 adults for all inferential analyses. The leading reasons for initial hesitancy were lack of understanding of the immediate protective role of PI (199/585, 34%), belief that vaccination alone was sufficient (194/585, 33%), and high cost (178/585, 30%). During the same clinical encounter, 320/585 adults (55%) chose to receive PI. Awareness of the early protection gap (aOR 2.63, 95% CI 1.75–3.98, *p* < 0.001) and bachelor’s degree or higher (aOR 1.61, 95% CI 1.07–2.43, *p* = 0.023) were positively associated with the decision to receive PI, whereas head/face wounds were negatively associated (aOR 0.53, 95% CI 0.30–0.92, *p* = 0.026). In reason-specific models, lack of knowledge about immediate protection was associated with higher odds of choosing PI (aOR 2.43, 95% CI 1.62–3.68, BH-FDR *p* < 0.001), whereas high cost was associated with lower odds (aOR 0.41, 95% CI 0.27–0.62, BH-FDR *p* < 0.001). Conclusion: Hesitancy toward and refusal of PI were observed among previously unvaccinated adults with Category III rabies exposure, yet initial hesitation or refusal is not irreversible. Early protection gap awareness was positively associated with PI choice, whereas cost-related concern was negatively associated. Clear explanation of protection timing and attention to cost-related barriers may support informed PI treatment decisions.

## 1. Introduction

Rabies is an acute, progressive, and almost invariably fatal viral encephalomyelitis caused by neurotropic viruses of the genus Lyssavirus within the family Rhabdoviridae [1]. Epidemiologically, rabies remains a major global public health concern, with an estimated 59,000 human deaths annually, predominantly occurring in Asia and Africa [2]. Comprehensive reviews further highlight its persistent epidemiological, diagnostic, and control challenges [3].

In the People’s Republic of China, rabies continues to pose a substantial public health challenge, consistently ranking among the top five notifiable infectious diseases in terms of mortality. It is estimated that approximately 40 million individuals in China experience animal bites or scratches annually, representing potential rabies exposures. Of these, around 10–12 million individuals receive PEP, resulting in substantial direct healthcare expenditures of approximately CNY 2–3 billion (equivalent to USD 0.28–0.42 billion, at an exchange rate of CNY 1 = USD 0.14, based on the average annual exchange rate of the People’s Bank of China in 2025) per year [4].

Against the background of China’s government-led, multisectoral One Health strategy [5], reported human rabies cases in mainland China declined steadily from 2048 in 2010 to a historic low of 122 in 2023 before increasing to 167 in 2024; the publicly reported annual count increased further to 244 in 2025 [6,7]. This pattern indicates a marked overall decline over the past decade, followed by two consecutive years of low-level resurgence after the historic low in 2023. Substantial geographic disparities remain, with high-risk foci concentrated primarily in rural counties of southwestern and central China [7,8,9].

According to the WHO 2018 position paper and expert consultation, PEP is the only effective intervention for preventing rabies after potential exposure and consists of wound management, active immunization with rabies vaccine, and PI when indicated [10,11]. Category III exposure comprises single or multiple transdermal bites or scratches, contamination of mucous membranes or broken skin with saliva from animal licks, or direct exposure to bats. In China, PEP is primarily administered in hospital emergency departments and designated rabies PEP clinics. Multiple licensed vaccines are routinely available for clinical PEP, most commonly human diploid cell vaccine and purified Vero cell rabies vaccine. Two classes of PI biologics are approved in China: HRIG and RMAb [12]. Costs vary by product: a complete PEP vaccination series costs approximately USD 40–210; HRIG is typically priced at USD 19–26 per 10 kg of body weight, whereas RMAb generally costs USD 58–87 per 10 kg of body weight. These products are largely paid out of pocket in routine outpatient PEP practice.

Per international rabies guidelines, passive immunization is indicated for Category III exposure in previously unvaccinated individuals. It delivers immediate neutralizing antibodies both locally at the wound site and systemically, bridging the early protection gap prior to the onset of vaccine-induced active immunity [13]. Breakthrough rabies infections after PEP have been consistently associated with suboptimal prophylaxis, most notably failures attributable to omitted or improperly delivered passive immunization [14]. Despite well-established guideline recommendations, real-world uptake of passive immunization remains suboptimal in multiple clinical settings, including mainland China. This underuse is likely multifactorial, stemming from limited understanding of its mechanistic role in PEP, the incremental out-of-pocket financial burden, and additional contextual barriers [12,15].

To address this evidence gap, we conducted a multicenter cross-sectional questionnaire study among previously unvaccinated patients with Category III rabies exposure who initially hesitated to receive or declined physician-recommended PI. The study objectives were to characterize this cohort, describe self-reported reasons for initial PI hesitancy or refusal, quantify PI treatment decisions recorded during the questionnaire encounter, identify factors associated with those decisions among adult respondents, and describe HRIG versus RMAb selection among adults who chose PI.

## 2. Methods

### 2.1. Study Design and Setting

This multicenter cross-sectional questionnaire study was conducted between 28 March and 21 June 2025 at 12 rabies PEP clinics or emergency departments spanning 12 provinces in mainland China, with one site selected per province (Supplementary Figure S1). The study sites were purposively selected to ensure broad geographic coverage, adequate PEP patient volume, and feasibility of standardized data collection. All sites provided guideline-based management for Category III rabies exposure and routinely offered both HRIG and RMAb. Before enrollment began, investigators from all sites completed the same centralized online training session covering eligibility screening, informed consent, questionnaire administration, contemporaneous recording of treatment decisions, medical chart review, and data-quality checks. This study is reported in accordance with the STROBE checklist statement.

### 2.2. Participants

Eligible participants were previously unvaccinated patients presenting to a study site after an index animal exposure that was clinically classified as Category III. Additional inclusion criteria were a clinician-confirmed indication for PI for that exposure, initial hesitation toward or declination of recommended PI, and ability to provide written informed consent. Patients unable to complete the questionnaire because of communication impairment or other limitations and patients who declined study participation were excluded.

The study protocol was approved by the Biomedical Research Ethics Committee of Peking University First Hospital (approval no. 2025R0333-0002). Written informed consent was obtained from all adult participants (aged ≥ 18 years) and from parents or legal guardians of minor participants (aged < 18 years). Age-appropriate assent from minors was acquired where applicable.

For minors, a parent or legal guardian completed decision-related, knowledge-related, socioeconomic, and attitude items, while the minor’s age, sex, and exposure characteristics were recorded.

### 2.3. Questionnaire Design

This study used an 18-item structured questionnaire developed from relevant clinical guidelines and routine clinical practice in rabies PEP. Domain experts in clinical medicine and public health reviewed the questionnaire for medical accuracy, content relevance, and wording clarity. Before formal data collection, the questionnaire was pilot-tested in multiple rounds with five eligible patients in clinical settings to assess item clarity, comprehensibility, response options, completion time, and feasibility and was refined according to their feedback.

The questionnaire covered eight domains: (1) sociodemographic characteristics; (2) exposure and wound characteristics; (3) comorbidity or special health condition; (4) awareness that vaccine-induced active immunity develops with a time lag and that PI provides immediate local neutralizing protection during the early protection gap; (5) reasons for initial PI hesitancy or refusal; (6) previous sources of rabies prevention knowledge and preferred communication channels; (7) the PI treatment decision recorded in Q17 during the same PEP encounter; and (8) HRIG or RMAb selection in Q18 among respondents who chose PI.

Awareness of the early protection gap was assessed with Q13, a single factual knowledge item asking whether respondents knew that rabies vaccination takes 7–10 days to confer protective immunity and that PI provides immediate protection during this interval (Supplementary Material S1). Factor analysis and internal-consistency testing were not applicable to this single-item measure. Test–retest reliability was not assessed because the questionnaire was not administered repeatedly to the same respondents.

Questions on reasons for PI hesitancy and knowledge acquisition channels allowed multiple responses. All questionnaire items were mandatory except those on exposure and wound characteristics and comorbidity or special health condition. The questionnaire was developed and administered in Mandarin Chinese (Putonghua); an English translation is provided in Supplementary Material S1.

### 2.4. Data Collection

Trained physician investigators administered the structured questionnaire to respondents at each site during the PEP encounter. Throughout the questionnaire completion process, investigators were available to address respondents’ queries in real time and provide detailed information regarding the clinical benefits of rabies vaccination and PI to facilitate informed acceptance of the recommended passive immunization regimen. The Q17 response recorded whether the patient chose PI during that encounter; for patients choosing PI, Q18 recorded the selected product, and the decision proceeded directly into the site’s prescribing and administration workflow. Exposure characteristics, wound characteristics, and the reported comorbidity or special health condition were verified against the medical chart before analysis.

Medical staff at participating sites conducted a telephone follow-up at least six months after questionnaire completion to ascertain vital status and screen for clinical rabies. During the follow-up, the site staff cross-checked rabies case notifications received from local or provincial Centers for Disease Control and Prevention against the study roster and reported any confirmed match to the coordinating center.

### 2.5. Sample Size

Sample size was calculated using the standard formula for cross-sectional studies, with a two-sided significance level (α) of 0.05, 5% absolute precision, and a conservative expected proportion of 0.50 because national surveillance data on PI hesitancy were unavailable. The minimum required sample size was 385; after allowing for 20% invalid responses, the target was 481.

### 2.6. Variable Definitions and Coding

The PI treatment decision recorded in Q17 during the same PEP encounter was coded 0 for declining PI and 1 for choosing PI. Among adults who chose PI, Q18 recorded selection of HRIG or RMAb. Explanatory variables covered sociodemographic characteristics, wound and exposure characteristics, animal-related factors, comorbidity or special health condition, awareness of the early protection gap, hesitancy reasons, and study site macro-region.

All inferential analyses were restricted to adults. Age was summarized as both continuous and categorical and entered into the multivariable models as a standardized continuous variable (1 SD = 10.98 years). Education, monthly household income, occupation, and the remaining questionnaire variables were grouped as specified in Supplementary Table S1, which lists the original response options, category-combination rules, analysis categories, and reference categories.

For clinical and animal-related variables, head/face wound was coded as present or absent, animal ownership as owned or stray, animal vaccination status as vaccinated, not vaccinated, or unknown, and comorbidity or special health condition as present or absent. Early protection gap awareness was coded as aware or unaware. For every analyzed variable, ‘unknown’ denoted a recorded response that the relevant information was not known, whereas missing denoted the absence of a recorded response or value. No values were missing for variables included in the adult analyses.

The study site provinces were grouped into four macro-regions: North (Beijing, Hebei, and Shanxi), East (Anhui, Jiangsu, Jiangxi, and Shandong), Central & Southwest (Chongqing, Henan, and Hubei), and South (Guangdong and Guangxi). This variable was used to account for differences among participating sites and was not intended for population-level regional inference.

### 2.7. Statistical Analysis

All statistical analyses were performed using R version 4.5.3 (R Core Team, R Foundation for Statistical Computing, Vienna, Austria) [16] and the following packages: tidyverse, readr, janitor, skimr, ggplot2, scales, gtsummary, flextable, officer, broom, car, binom, viridis, tableone, epiDisplay, sandwich, lmtest, and ragg. Continuous variables were summarized as mean ± SD and categorical variables as counts and percentages. PI treatment decision groups were compared using the Wilcoxon rank-sum test for continuous variables and Pearson’s chi-square test for categorical variables. Chi-square *p* values were obtained by Monte Carlo simulation with 10,000 replicates when expected cell counts were small; a fixed random seed ensured reproducibility. Province-specific PI acceptance rates and 95% CIs were calculated with the Wilson method.

Factors associated with the PI treatment decision were evaluated with a prespecified full multivariable binary logistic regression model (N = 585). The covariates were sex, standardized age, education, monthly household income, occupation, number of wounds, head/face wound, animal ownership, animal vaccination status, comorbidity or special health condition, awareness of the early protection gap, and macro-region. The results are reported as adjusted odds ratios (aORs) with 95% CIs, and full model estimates are provided in Supplementary Table S2. Generalized variance inflation factors were assessed using the empirical threshold GVIF^(1/[2 × Df]) > 3.

Reason-specific logistic regression models evaluated associations between each of the eight reported reasons for initial PI hesitancy or refusal and the PI treatment decision. Model 1 adjusted for age, monthly household income, and macro-region; Model 2 additionally adjusted for awareness of the early protection gap. The Benjamini–Hochberg false discovery rate correction was applied to the eight reason-specific comparisons within each model. Sensitivity analyses included an AIC-based stepwise model (Supplementary Table S3) and the prespecified full model using province-clustered heteroskedasticity-consistent robust SEs (Supplementary Table S4); both used the 585-adult analytic population. An exploratory multivariable binary logistic regression model among the 320 adults who chose PI evaluated factors associated with RMAb selection, with HRIG as the reference. The covariates were sex, standardized age, education, monthly household income, head/face wound, animal ownership, animal vaccination status, comorbidity or special health condition, and macro-region.

All tests were two-sided, with *p* < 0.05 considered statistically significant. For the reason-specific analyses, both the nominal and BH-FDR-adjusted *p* values are reported.

## 3. Results

### 3.1. Study Population and PI Treatment Decision

During the study period, 5068 previously unvaccinated patients with Category III rabies exposure presented across the 12 study sites and had a clinical indication for PI. Of these, 2866 (57%) initially hesitated to receive or declined PI, and 617 completed the questionnaire. Over the 6-month follow-up period, none of the 617 questionnaire respondents developed clinical rabies.

The review of the sociodemographic responses provided for minors, including education, occupation, and income, showed that the questionnaire did not clearly specify whether these items referred to the minor patient or to the parent or legal guardian making the treatment decision. Because it was unclear whose information these responses represented, the 32 questionnaires for minors were excluded from all analyses involving sociodemographic variables. These analyses were conducted using the 585-adult dataset in which the patient and the treatment decision-maker were the same person. Both the full questionnaire cohort (N = 617) and the adult dataset (N = 585) exceeded the target sample size of 481. Participant flow is shown in Supplementary Figure S2, and adult respondent counts by study site are shown in Supplementary Figure S3.

The mean age of adult respondents was 37 ± 11 years; 289 (49%) were female, and 296 (51%) were male (Table 1).

During questionnaire completion, 320/585 adults (55%) reported changing their decision and expressed willingness to receive PI, whereas 265/585 (45%) maintained their refusal. The prescription record review confirmed no discrepancies between patients’ questionnaire responses and actual clinical management. Province-specific PI acceptance rates are shown in Supplementary Figure S4. Unadjusted comparisons between the two groups showed differences in age, age category, education, macro-region, head/face wound, and awareness of the early protection gap (Table 1).

Adults who chose PI were younger than those who declined it (mean age 36 ± 10 vs. 38 ± 12 years, *p* = 0.035); the age category distribution also differed (*p* = 0.013). The adult age distribution is shown in Supplementary Figure S5. Bachelor’s degree or higher was more frequent among adults who chose PI (166/320, 52%) than among those who declined it (106/265, 40%; *p* = 0.024). Macro-region composition also differed (*p* < 0.001): the accepted-PI group was concentrated in Central & Southwest (118/320, 37%) and East (112/320, 35%), whereas the declined-PI group drew mainly from North (80/265, 30%) and South (79/265, 30%).

Head/face wounds were more frequent among adults who declined PI (40/265, 15%) than among those who chose it (30/320, 9.4%; *p* = 0.046). Monthly household income showed a borderline group difference (*p* = 0.068). Occupation, number of wounds, animal species, animal ownership, animal vaccination status, and the presence of a comorbidity or special health condition did not differ significantly between treatment decision groups. When animal vaccination status was recoded as vaccinated versus not vaccinated or unknown, no association with the treatment decision was observed in the unadjusted comparison (*p* = 0.412) or the adjusted model (aOR 1.29, 95% CI 0.85–1.96, *p* = 0.240).

Awareness of the early protection gap was reported by 315/585 adults (54%). Awareness was higher among adults who chose PI (197/320, 62%) than among those who declined it (118/265, 45%; *p* < 0.001).

### 3.2. Reasons for Initial PI Hesitancy or Refusal

**Figure 1:**
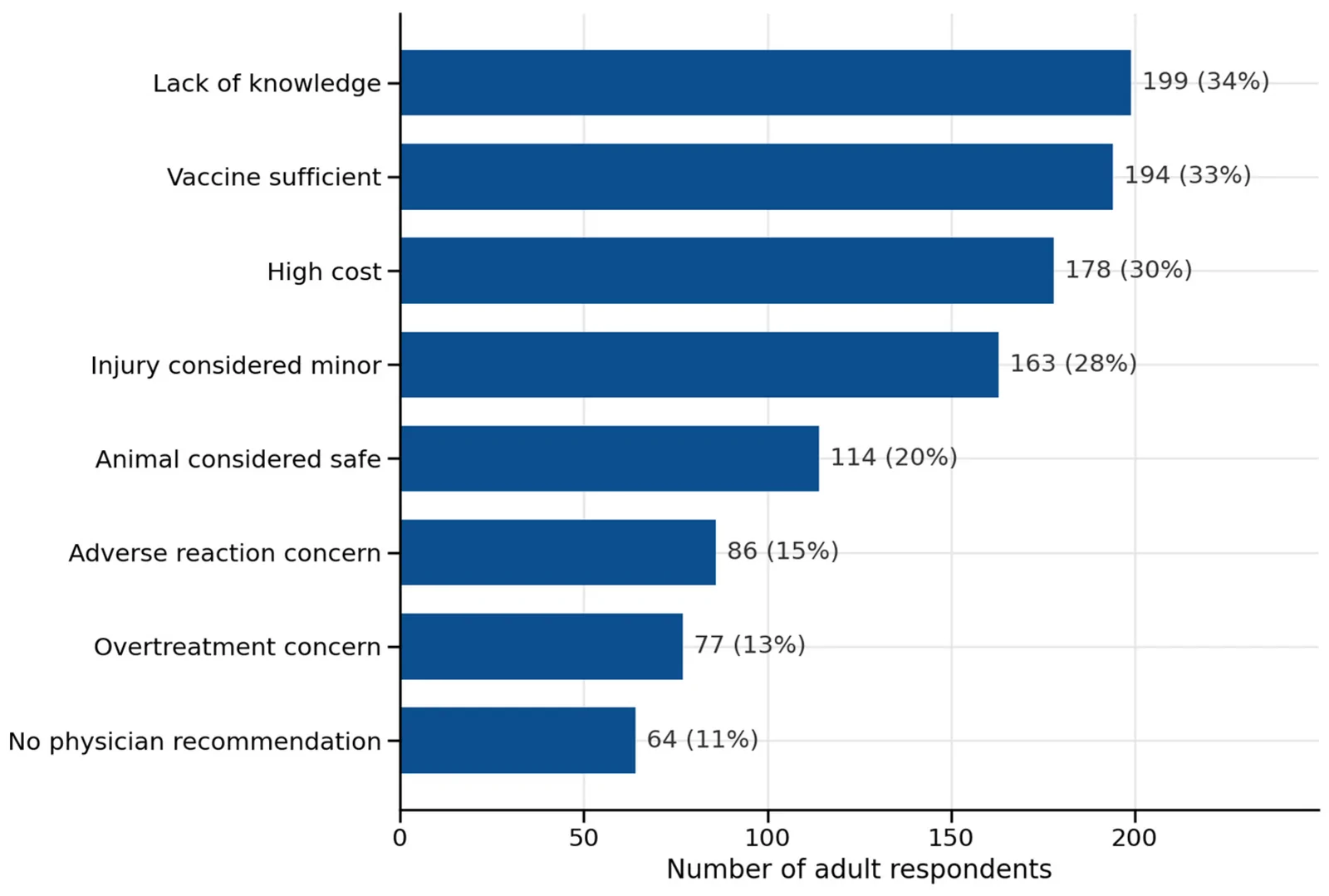
Reasons for initial PI hesitancy or refusal among 585 adult respondents. Multiple responses were allowed; percentages use all 585 adults as the denominator.

The most frequently selected reasons were lack of knowledge about the immediate protective role of PI (199/585, 34%), belief that vaccination alone was sufficient (194/585, 33%), high cost (178/585, 30%), and perception that the injury was not serious (163/585, 28%). Other reasons were believing that the animal was safe (114/585, 20%), concern about adverse reactions (86/585, 15%), concern about overtreatment (77/585, 13%), and lack of physician recommendation (64/585, 11%) (Figure 1).

Among the 114 adults who reported that the animal was considered safe, 73 (64.0%) reported a vaccinated animal, compared with 248/471 (52.7%) who did not report this reason (*p* = 0.0019). Among the 64 adults who reported no physician recommendation, 23 (35.9%) reported a vaccinated animal, compared with 298/521 (57.2%) who did not report this reason (*p* = 0.0040) (Supplementary Table S5). Adverse reaction concern was reported by 12/70 adults (17.1%) with a head/face wound and 74/515 (14.4%) without a head/face wound (*p* = 0.539); overtreatment concern was reported by 7/70 (10.0%) and 70/515 (13.6%), respectively (*p* = 0.404) (Supplementary Table S6).

Exploratory co-occurrence analysis identified recurring pairs of reasons for PI hesitancy or refusal. The most frequent pairs were perceived non-severe injury with belief that vaccination alone was sufficient (n = 78), perceived non-severe injury with perceived animal safety (n = 63), belief that vaccination alone was sufficient with perceived animal safety (n = 59), and belief that vaccination alone was sufficient with lack of awareness of PI’s early protective effect (n = 54) (Supplementary Figure S6).

### 3.3. Factors Associated with the PI Treatment Decision

**Figure 2:**
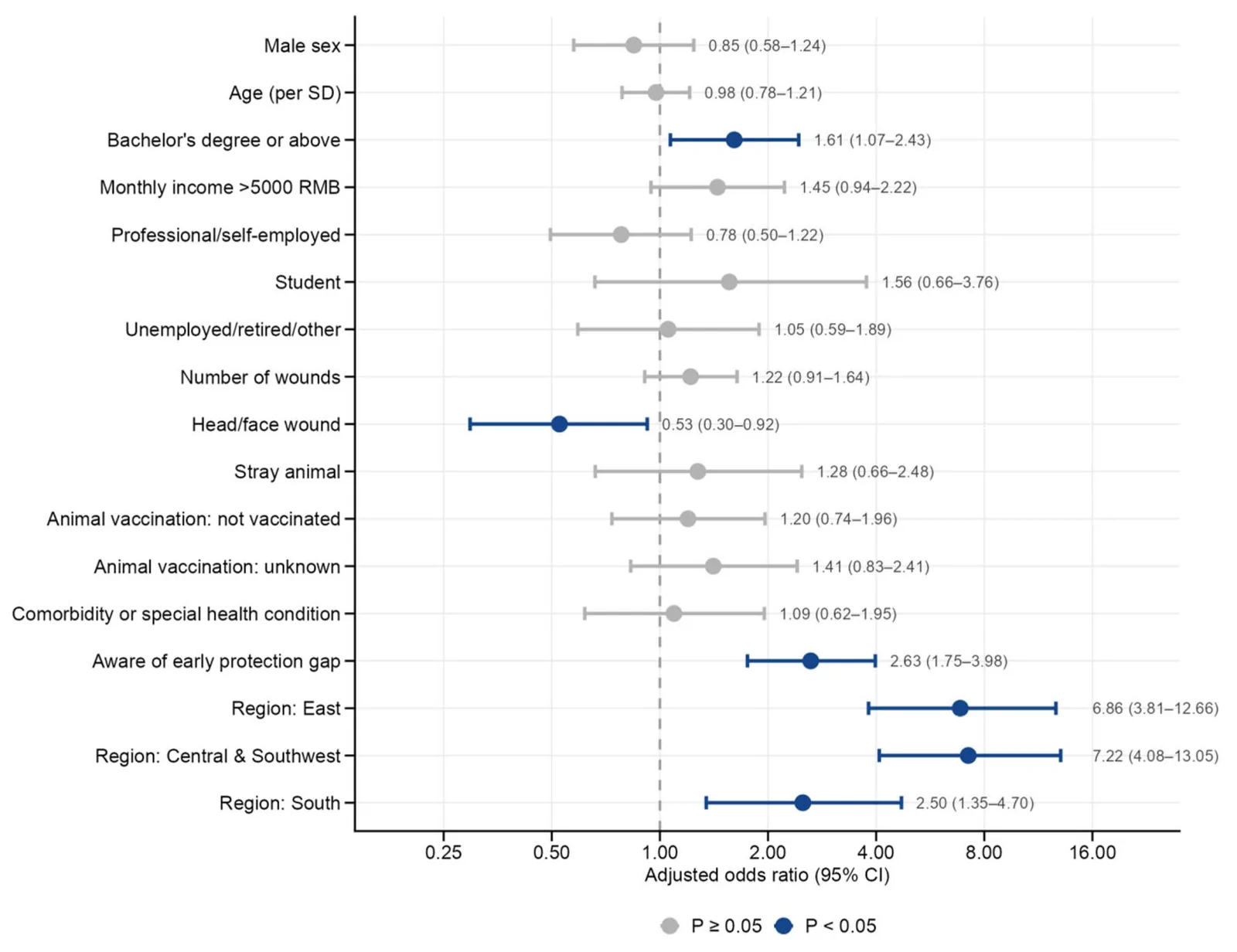
Factors associated with the PI treatment decision among 585 adult respondents. aORs and 95% CIs were estimated using the prespecified full multivariable logistic regression model. Age was standardized, with 1 SD = 10.98 years. Reference categories are provided in Supplementary Table S1.

In the prespecified full multivariable model of 585 adults, the PI treatment decision was associated with education, head/face wound, awareness of the early protection gap, and macro-region (Figure 2; Supplementary Table S2). Adults with bachelor’s degree or higher had greater odds of choosing PI (aOR 1.61, 95% CI 1.07–2.43, *p* = 0.023).

Awareness of the early protection gap was associated with greater odds of choosing PI (aOR 2.63, 95% CI 1.75–3.98, *p* < 0.001), and the corresponding descriptive association is shown in Supplementary Figure S7. A head/face wound was associated with lower odds of choosing PI (aOR 0.53, 95% CI 0.30–0.92, *p* = 0.026).

Compared with North, the odds of choosing PI were higher in East (aOR 6.86, 95% CI 3.81–12.66, *p* < 0.001), Central & Southwest (aOR 7.22, 95% CI 4.08–13.05, *p* < 0.001), and South (aOR 2.50, 95% CI 1.35–4.70, *p* = 0.004). Sex, age, occupation, number of wounds, animal ownership, animal vaccination status, and the presence of a comorbidity or special health condition were not statistically significant in the full model.

The AIC-based sensitivity model retained bachelor’s degree or higher, head/face wound, stray animal exposure, awareness of the early protection gap, and macro-region (Supplementary Table S3). In the prespecified full model with province-clustered robust SEs, head/face wound, awareness of the early protection gap, East and Central & Southwest remained statistically significant, while the South estimate was attenuated (Supplementary Table S4).

### 3.4. Reasons for Initial PI Hesitancy or Refusal and the PI Treatment Decision

Two reason-specific logistic regression models evaluated associations between the eight reasons and the PI treatment decision among 585 adults. The BH-FDR correction was applied to the eight comparisons in each model (Table 2). In Model 1, lack of knowledge about immediate protection was associated with greater odds of choosing PI (aOR 2.43, 95% CI 1.62–3.68, *p* < 0.001, BH-FDR *p* < 0.001), while high cost was associated with lower odds (aOR 0.41, 95% CI 0.27–0.62, *p* < 0.001, BH-FDR *p* < 0.001). Belief that vaccination alone was sufficient had a nominal association (aOR 1.58, 95% CI 1.05–2.38, *p* = 0.029) that did not remain significant after correction (BH-FDR *p* = 0.076).

After additional adjustment for awareness of the early protection gap in Model 2, lack of knowledge about immediate protection remained positively associated with choosing PI (aOR 2.65, 95% CI 1.75–4.05, *p* < 0.001, BH-FDR *p* < 0.001), and high cost remained negatively associated (aOR 0.49, 95% CI 0.32–0.75, *p* = 0.001, BH-FDR *p* = 0.004). The estimate for belief that vaccination alone was sufficient was attenuated (aOR 1.42, 95% CI 0.94–2.16, *p* = 0.099, BH-FDR *p* = 0.263). The complete results are provided in Supplementary Table S7 for Model 1 and Supplementary Table S8 and Supplementary Figure S8 for Model 2.

### 3.5. Information Sources and Future Communication Preferences

**Figure 3:**
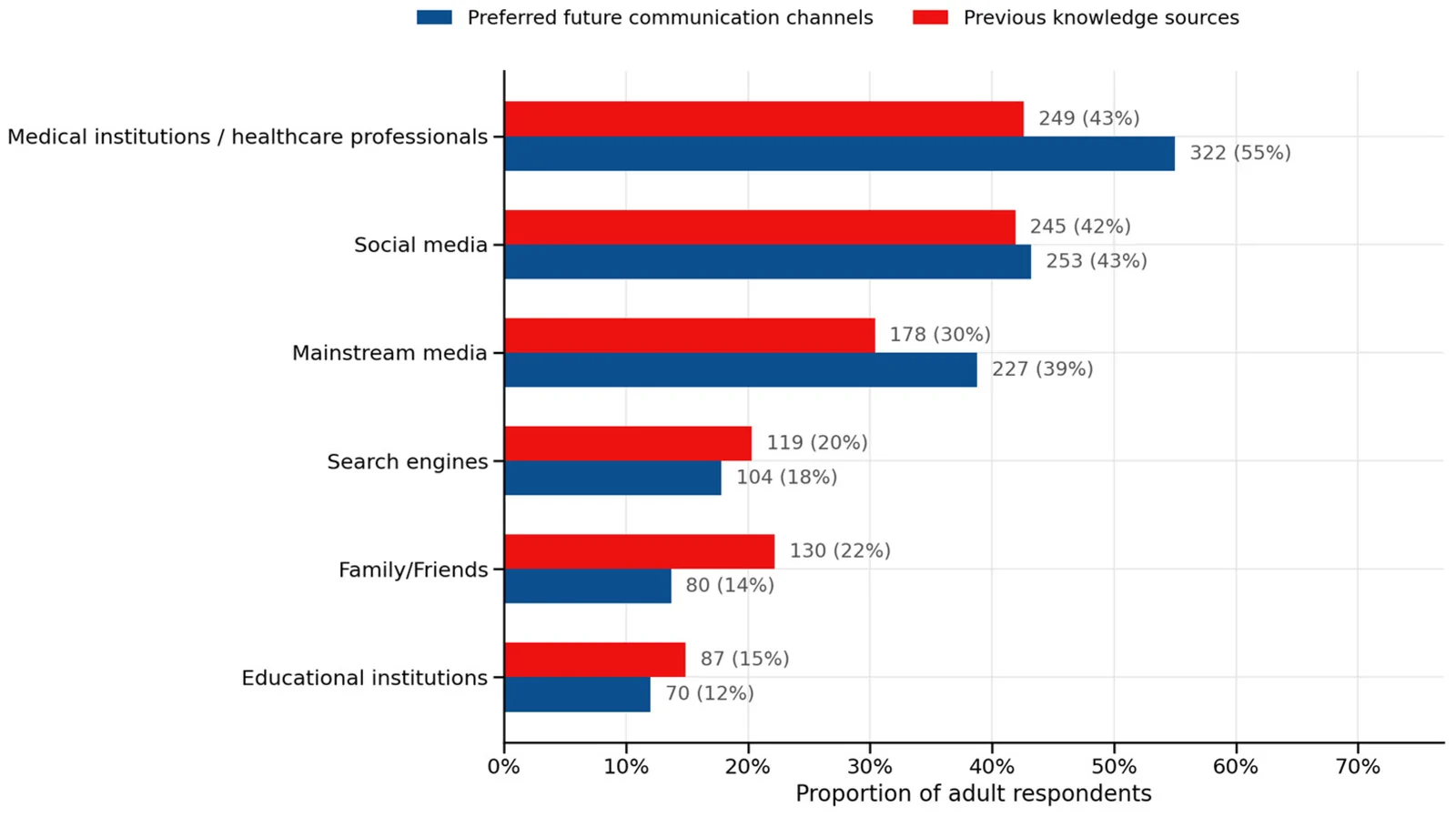
Previous knowledge sources and preferred future communication channels among 585 adult respondents. Multiple responses were allowed; percentages use all 585 adults as the denominator.

The most frequently reported previous sources of rabies prevention knowledge were medical institutions or health professionals (249/585, 43%) and social media (245/585, 42%), followed by mainstream media (178/585, 30%), family or friends (130/585, 22%), search engines (119/585, 20%), and educational institutions (87/585, 15%). A further 147/585 adults (25%) reported that they had not previously known about rabies prevention. Preferred future communication channels were medical institutions or health professionals (322/585, 55%), social media (253/585, 43%), and mainstream media (227/585, 39%) (Figure 3).

In a descriptive supplementary analysis, PI acceptance was highest among respondents who selected educational institutions as an effective future communication channel (60/70, 85.7%; 95% CI 74.8–92.6%), followed by mainstream media (148/227, 65.2%; 95% CI 58.6–71.3%), social media (152/253, 60.1%), medical institutions or health professionals (190/322, 59.0%), search engines (59/104, 56.7%), and family or friends (43/80, 53.8%) (Supplementary Figure S9).

### 3.6. Exploratory PI Product Selection Among Adults Who Chose PI

Among the 320 adults who chose PI, 176 (55%) selected HRIG, and 144 (45%) selected RMAb (Supplementary Figure S10). The prescription record review confirmed no discrepancies between patients’ questionnaire responses and actual clinical management. RMAb selection was descriptively more frequent among respondents with higher monthly household income and a bachelor’s degree or higher (Supplementary Figures S11 and S12). In an exploratory multivariable analysis, bachelor’s degree or higher, the presence of a comorbidity or special health condition, and macro-region were associated with RMAb rather than HRIG selection (Supplementary Table S9).

## 4. Discussion

Vaccine hesitancy is defined by the WHO Strategic Advisory Group of Experts as a delay in acceptance or refusal despite the availability of vaccination services [17]. For rabies PEP, previous Chinese studies have documented refusal, underuse, or incomplete uptake of RIG, supporting the clinical relevance of hesitancy toward guideline-recommended PI [18,19,20]. Our multicenter study focused on previously unvaccinated adults with Category III exposure who initially hesitated to receive or declined physician-recommended PI. More than half of the enrolled adults chose PI during the same PEP encounter. Awareness of the early protection gap was associated with choosing PI, whereas reporting high cost as a reason for initial hesitancy was associated with a lower likelihood of choosing PI. The overall pattern suggests that some initial PI hesitancy can be reconsidered during clinical care and highlights the need to address knowledge-related uncertainty and cost-related concern in different ways.

The reasons reported for initial hesitancy help explain why these two findings call for different responses. The respondents described limited understanding of PI’s immediate protective role, belief that vaccination alone was sufficient, low perceived injury severity or animal risk, and cost. Similar combinations of knowledge, risk perception, confidence, and access have been described in research on rabies prevention and vaccine hesitancy [21,22,23,24,25]. Knowledge about protection timing has a clear clinical basis: vaccine-induced active immunity takes time to develop, while PI supplies immediate neutralizing protection during this interval. WHO guidance therefore treats wound care, vaccination, and PI as complementary components of Category III PEP, and reviews of fatal breakthrough rabies have linked some failures to incomplete or improperly delivered PI [10,11,14]. In our analyses, early protection gap awareness was associated with choosing PI, and the estimate for belief in vaccine-only sufficiency weakened after awareness was added to the model. This pattern makes protection timing a practical focus for explanation. Clinicians can state that vaccine initiates active immunity and PI protects the patient while that immunity develops, using clear language about timing, benefit, uncertainty, and personal relevance as recommended in health-literacy and shared-decision-making research [26,27]. Awareness and treatment choice were recorded during the same encounter, so the direction of this association remains uncertain; patients already inclined to choose PI may also have paid closer attention to the explanation.

Cost-related concern had a different practical implication. Adults who reported high cost as a reason for initial PI hesitancy were less likely to choose PI after adjustment for age, monthly household income, macro-region, and early protection gap awareness. The questionnaire measured self-reported monthly household income and did not assess affordability; the finding therefore reflects perceived economic burden and cost-related hesitancy. Studies in other rabies-endemic settings have likewise identified treatment cost as an influence on PEP use [28,29]. These findings support discussing the expected cost of PI clearly when treatment options are explained. Cost barriers cannot be addressed by individual clinicians alone and require public health policy support.

Adults with head/face wounds were less likely to choose PI even though these exposures generally warrant high clinical priority. The mechanism underlying this association remains uncertain because the questionnaire did not assess concerns specific to treatment around the head or face. This finding may reflect a mismatch between clinical risk and patients’ subjective risk perception. Risk perception research shows that emotional and contextual cues can influence how people interpret clinical danger [23,30]. Adverse reaction and overtreatment concerns were reported at similar frequencies among adults with and without head/face wounds, offering no clear explanation for the association. During PEP care, clinicians should explain the importance of head and facial exposures and ask about the concerns shaping the treatment decision.

This study observed that the likelihood of receiving PI differed among adult respondents across macro-regions. However, because only one study site was included per province, geographic region was closely confounded with study site. This finding should therefore be interpreted as between-site heterogeneity rather than evidence of regional differences in patient attitudes or risk perception. To further improve acceptance of PI in future work, in addition to refining patient education content, it may also be warranted to conduct comparative studies across study sites examining differences in risk communication, cost clarification, product counseling, and physician–patient communication workflows so as to identify replicable best practices.

Medical institutions and health professionals were also the most common previous source and the most preferred future channel for rabies prevention information. Communication research supports timely, specific, and usable information from trusted sources [31]. These findings reinforce the role of the PEP encounter: clinicians can explain why vaccine and PI are needed at different stages after exposure, identify the concern that remains after the explanation, and discuss treatment costs when they are a concern.

Product selection arose after a patient had chosen PI and therefore addressed a narrower question. Among these adults, HRIG and RMAb were selected in similar proportions. RMAb products such as SYN023 provide a clinically active alternative to HRIG [32,33]. The exploratory associations of product choice with education, the presence of a comorbidity or special health condition, and macro-region warrant further study with direct measurement of product price, availability, counseling, and prior knowledge.

This study has three main limitations. First, the sample was drawn by convenience from 12 PEP facilities, one per province, and 617 of the 2866 patients who initially hesitated to receive or declined PI completed the questionnaire; inferential analyses included 585 adults. The findings therefore describe enrolled adults who sought care and received a recommendation for PI and may not represent nonparticipants, minors, or people who never attended a PEP facility. Because information on nonparticipants was unavailable, self-selection may also have influenced the observed associations, although the direction and magnitude of this influence cannot be determined. Broader prospective recruitment across multiple sites within provinces, together with basic data on nonparticipants where feasible, would improve generalizability. Second, awareness of the early protection gap and the PI treatment decision were assessed during the same clinical encounter, so the direction of the observed association cannot be determined. Third, the questionnaire did not capture detailed information on clinic communication, actual treatment costs, or the circumstances surrounding each exposure. These unmeasured factors may have contributed to differences in PI decisions across participating sites and to the association observed for head/face wounds. Future studies should collect these data prospectively and could link patient records with animal vaccination, post-bite observation, and animal testing information within the One Health framework. In addition, the study did not use a standardized educational intervention protocol. The questionnaire included information about PI. The PI treatment decisions observed during the PEP visit were therefore made after participants received this information and may differ from decisions made during routine, less structured clinical communication.

## 5. Conclusions

In conclusion, hesitancy toward and refusal of PI were observed among previously unvaccinated adults with Category III rabies exposure, yet initial hesitation or refusal is not irreversible. More than half of the respondents changed their minds and accepted PI during the same PEP encounter. The associations with early protection gap awareness and cost-related concern identify two practical priorities for PEP care: explain why vaccine and PI are needed at different stages after exposure, and discuss treatment costs clearly when patients express cost-related concerns. These findings come from a cross-sectional, facility-based sample of enrolled adults and require confirmation in broader prospective studies. Future studies should evaluate whether standardized explanations of protection timing and clear communication about treatment costs improve appropriate PI uptake after high-risk exposure.

## Figures and Tables

**Table 1:** Characteristics of previously unvaccinated adult respondents initially hesitant about or declining passive immunization, by PI treatment decision (N = 585).

Characteristic	Overall N = 585 ^1^	PI Treatment Declined n = 265 ^1^	PI Treatment Accepted n = 320 ^1^	*p*-Value ^2^
Sex				0.118
Female	289 (49%)	121 (46%)	168 (53%)	
Male	296 (51%)	144 (54%)	152 (48%)	
**Age** **, years**	**37 ± 11**	**38 ± 12**	**36 ± 10**	**0.035**
**Age group, years**				**0.013**
**18–30**	**175 (30%)**	**76 (29%)**	**99 (31%)**	
**31–45**	**296 (51%)**	**122 (46%)**	**174 (54%)**	
**46–60**	**92 (16%)**	**53 (20%)**	**39 (12%)**	
**>60**	**22 (4%)**	**14 (5%)**	**8 (3%)**	
Occupation				0.466
Manual worker/Farmer	203 (35%)	99 (37%)	104 (33%)	
Professional/Self-employed	257 (44%)	116 (44%)	141 (44%)	
Student	40 (7%)	15 (6%)	25 (8%)	
Unemployed/Retired/Other	85 (15%)	35 (13%)	50 (16%)	
**Education level**				**0.024**
**Junior high school or below**	**94 (16%)**	**52 (20%)**	**42 (13%)**	
**High school**	**86 (15%)**	**43 (16%)**	**43 (13%)**	
**Associate degree**	**133 (23%)**	**64 (24%)**	**69 (22%)**	
**Bachelor’s degree or higher**	**272 (46%)**	**106 (40%)**	**166 (52%)**	
Monthly household income, CNY (USD)				0.068
<3000 (<420)	152 (26%)	80 (30%)	72 (23%)	
3001–5000 (420–700)	153 (26%)	71 (27%)	82 (26%)	
5001–10,000 (700–1400)	181 (31%)	69 (26%)	112 (35%)	
>10,000 (>1400)	99 (17%)	45 (17%)	54 (17%)	
**Macro-region**				**<0.001**
**North**	**115 (20%)**	**80 (30%)**	**35 (11%)**	
**East**	**172 (29%)**	**60 (23%)**	**112 (35%)**	
**Central & Southwest**	**164 (28%)**	**46 (17%)**	**118 (37%)**	
**South**	**134 (23%)**	**79 (30%)**	**55 (17%)**	
Number of wounds				0.776
1 wound	425 (73%)	189 (71%)	236 (74%)	
2–3 wounds	112 (19%)	54 (20%)	58 (18%)	
>3 wounds	48 (8%)	22 (8%)	26 (8%)	
**Head/face wound**				**0.046**
**No**	**515 (88%)**	**225 (85%)**	**290 (91%)**	
**Yes**	**70 (12%)**	**40 (15%)**	**30 (9%)**	
Injuring animal species				0.235
Dog	314 (54%)	139 (52%)	175 (55%)	
Cat	226 (39%)	110 (42%)	116 (36%)	
Other	45 (8%)	16 (6%)	29 (9%)	
Animal source				0.169
Owned animal	517 (88%)	240 (91%)	277 (87%)	
Stray animal	68 (12%)	25 (9%)	43 (13%)	
Animal vaccination status				0.228
Vaccinated	321 (55%)	140 (53%)	181 (57%)	
Not vaccinated	129 (22%)	67 (25%)	62 (19%)	
Unknown ^3^	135 (23%)	58 (22%)	77 (24%)	
Comorbidity or special health condition				0.950
Absent	516 (88%)	233 (88%)	283 (88%)	
Present	69 (12%)	32 (12%)	37 (12%)	
**Awareness of early protection gap**				**<0.001**
**Unaware**	**270 (46%)**	**147 (55%)**	**123 (38%)**	
**Aware**	**315 (54%)**	**118 (45%)**	**197 (62%)**	

^1^ n (%); mean ± SD. ^2^ Wilcoxon rank-sum test for age; Pearson chi-square tests with Monte Carlo simulation (10,000 replicates) for categorical variables. ^3^ Throughout the analyses, ‘unknown’ denotes an explicitly recorded response that the relevant information was not known and is treated as an observed category; missing data denotes the absence of a recorded value. No values were missing for the variables reported in Table 1.

**Table 2:** Associations between reasons for initial PI hesitancy or refusal and the PI treatment decision among 585 adults. Model 1 adjusted for age, monthly household income, and macro-region; Model 2 additionally adjusted for awareness of the early protection gap. Bold values indicate BH-FDR-adjusted *p* < 0.05.

Initial Hesitancy Reason	Model 1: Core Adjustment			Model 2: Additional Knowledge Adjustment		
Initial Hesitancy Reason	aOR (95% CI)	*p* Value	BH-FDR *p* Value	aOR (95% CI)	*p* Value	BH-FDR *p* Value
**Lack of knowledge about immediate protection**	**2.43 (1.62–3.68)**	**<0.001**	**<0.001**	**2.65 (1.75–4.05)**	**<0.001**	**<0.001**
Vaccine deemed sufficient	1.58 (1.05–2.38)	0.029	0.076	1.42 (0.94–2.16)	0.099	0.263
**High cost**	**0.41 (0.27–0.62)**	**<0.001**	**<0.001**	**0.49 (0.32–0.75)**	**0.001**	**0.004**
Injury considered minor	0.82 (0.53–1.26)	0.361	0.578	0.78 (0.50–1.22)	0.280	0.449
Animal considered safe	1.45 (0.88–2.41)	0.146	0.291	1.38 (0.84–2.31)	0.207	0.414
Adverse reaction concern	0.98 (0.58–1.67)	0.951	0.951	1.03 (0.61–1.77)	0.904	0.947
Overtreatment concern	1.02 (0.58–1.82)	0.945	0.951	0.98 (0.55–1.76)	0.947	0.947
No physician recommendation	0.95 (0.52–1.75)	0.861	0.951	0.95 (0.51–1.77)	0.869	0.947

## Data Availability

The de-identified data supporting the findings of this study are available from the corresponding author upon reasonable request and subject to applicable institutional and ethical requirements.

## Supplementary Materials

The following supporting information can be downloaded at: https://www.mdpi.com/article/10.3390/tropicalmed11090238/s1, STROBE checklist; Supplementary Material S1: Questionnaire for patients refusing passive immunization. Supplementary Figure S1: Geographic distribution of 585 adult respondents across participating provinces. The map shows the enrolled study sites. Supplementary Figure S2: Participant flow from eligible Category III exposures to the adult analytic population. Supplementary Figure S3: Adult respondent count by study site (N = 585). Supplementary Figure S4: Province-specific PI acceptance rates among 585 adults. Points show observed proportions, error bars show Wilson 95% CIs, and point size reflects the province sample size. The dashed line shows the overall adult acceptance rate (320/585, 54.7%). Supplementary Figure S5: Age distribution of 585 adults, stratified by PI treatment decision. The dotted line marks age 18 years. Supplementary Figure S6: Co-occurrence of reported reasons for initial PI hesitancy or refusal among 585 adults. The triangle displays pairwise overlaps among the eight multiple-response reasons. Supplementary Figure S7: Awareness of the early protection gap and PI acceptance among 585 adults. Error bars show Wilson 95% CIs. Supplementary Figure S8: Reason-specific Model 2 for the PI treatment decision among 585 adults. The model adjusted for age, monthly household income, macro-region, and awareness of the early protection gap. Open red circles identify reasons that remained significant after BH-FDR correction. Supplementary Figure S9: PI acceptance rates by preferred future communication channel among adult respondents. Multiple channels could be selected; denominators therefore vary by channel. Error bars show Wilson 95% CIs, point size reflects the channel-specific denominator, and the dashed line shows the overall adult acceptance rate. Supplementary Figure S10: PI treatment decision among all 585 adults and PI product selection among the 320 adults who chose. Supplementary Figure S11: HRIG and RMAb selection by monthly household income among the 320 adults who chose PI. Supplementary Figure S12: HRIG and RMAb selection by educational attainment among the 320 adults who chose PI. Supplementary Table S1: Original questionnaire response options and coding used in adult analyses (N = 585). Supplementary Table S2: Prespecified full multivariable logistic regression model for the PI treatment decision among adults (N = 585). Supplementary Table S3: AIC-based adult sensitivity model for the PI treatment decision (N = 585). Supplementary Table S4: Prespecified adult model for the PI treatment decision with province-clustered robust standard errors (N = 585). Supplementary Table S5: Animal vaccination status among adults with and without two reported reasons for initial PI hesitancy or refusal (N = 585). Supplementary Table S6: Selected concerns by head/face wound status among adults (N = 585). Supplementary Table S7: Complete reason-specific Model 1 for the PI treatment decision without adjustment for early-protection-gap awareness (N = 585). Supplementary Table S8: Complete reason-specific Model 2 for the PI treatment decision with adjustment for early-protection-gap awareness (N = 585). Supplementary Table S9: Exploratory multivariable model of RMAb versus HRIG selection among adults who chose PI (N = 320).
