# Supplementary material for: Passive Immunization Hesitancy Among Rabies-Exposed Individuals in China: A Multicenter Cross-Sectional Study
Source: Trop Med Infect Dis. 2026 Aug 24;11(9):238. doi: 10.3390/tropicalmed11090238 (PMC13611066; doi:10.3390/tropicalmed11090238)
Supplement: Supplementary file 1 [file tropicalmed-11-00238-s001.zip › STROBE Checklist.pdf]

STROBE Statement—Checklist of items that should be included in reports of ***cross-sectional studies***

	Item No	Recommendation	Location in manuscript
**Title and abstract**	1	(*a*) Indicate the study's design with a commonly used term in the title or the abstract	Title; Abstract, Methods
		(*b*) Provide in the abstract an informative and balanced summary of what was done and what was found	Abstract, Background, Methods, Results, and Conclusion
**Introduction**			
Background/rationale	2	Explain the scientific background and rationale for the investigation being reported	Introduction, paragraphs 1-4
Objectives	3	State specific objectives, including any prespecified hypotheses	Introduction, final paragraph
**Methods**			
Study design	4	Present key elements of study design early in the paper	Methods, Study Design and Setting
Setting	5	Describe the setting, locations, and relevant dates, including periods of recruitment, exposure, follow-up, and data collection	Methods, Study Design and Setting
Participants	6	(*a*) Give the eligibility criteria, and the sources and methods of selection of participants	Methods, Participants
Variables	7	Clearly define all outcomes, exposures, predictors, potential confounders, and effect modifiers. Give diagnostic criteria, if applicable	Methods, Questionnaire Design; Variable Definitions and Coding; Statistical Analysis; Supplementary Table S1
Data sources/ measurement	8*	For each variable of interest, give sources of data and details of methods of assessment (measurement). Describe comparability of assessment methods if there is more than one group	Methods, Questionnaire Design; Data Collection; Variable Definitions and Coding; Supplementary Material S1
Bias	9	Describe any efforts to address potential sources of bias	Methods, Study Design and Setting; Questionnaire Design; Data Collection; Statistical Analysis; Discussion, Limitations
Study size	10	Explain how the study size was arrived at	Methods, Sample Size
Quantitative variables	11	Explain how quantitative variables were handled in the analyses. If applicable, describe which groupings were chosen and why	Methods, Variable Definitions and Coding; Statistical Analysis; Table 1; Supplementary Table S1
Statistical methods	12	(*a*) Describe all statistical methods, including those used to control for confounding	Methods, Statistical Analysis

		(*b*) Describe any methods used to examine subgroups and interactions	Methods, Statistical Analysis; reason-specific Models 1 and 2; adult sensitivity analyses
		(*c*) Explain how missing data were addressed	Methods, Data Collection; Variable Definitions and Coding
		(*d*) If applicable, describe analytical methods taking account of sampling strategy	Not applicable; no complex sampling weights were used. Methods, Study Design and Setting; Statistical Analysis
		(*e*) Describe any sensitivity analyses	Methods, Statistical Analysis; Supplementary Tables S3 and S4
**Results**			
Participants	13*	(a) Report numbers of individuals at each stage of study—eg numbers potentially eligible, examined for eligibility, confirmed eligible, included in the study, completing follow-up, and analysed	Results, Study Population and PI Treatment Decision; Supplementary Figure S12
		(b) Give reasons for non-participation at each stage	Results, Study Population and PI Treatment Decision; detailed reasons for non-completion were not collected
		(c) Consider use of a flow diagram	Supplementary Figure S12
Descriptive data	14*	(a) Give characteristics of study participants (eg demographic, clinical, social) and information on exposures and potential confounders	Results, Study Population and PI Treatment Decision; Table 1
		(b) Indicate number of participants with missing data for each variable of interest	Methods, Data Collection; Variable Definitions and Coding; Table 1 footnotes
Outcome data	15*	Report numbers of outcome events or summary measures	Results, Study Population and PI Treatment Decision; Table 1; Supplementary Figure S9
Main results	16	(*a*) Give unadjusted estimates and, if applicable, confounder-adjusted estimates and their precision (eg, 95% confidence interval). Make clear which confounders were adjusted for and why they were included	Results, Factors Associated With the PI Treatment Decision; Table 2; Figure 2; Supplementary Tables S2, S5, and S6
		(*b*) Report category boundaries when continuous variables were categorized	Methods, Variable Definitions and Coding; Table 1; Supplementary Table S1
		(*c*) If relevant, consider translating estimates of relative risk into absolute risk for a meaningful time period	Not applicable; the PI treatment decision was observed during the clinical encounter and was not a time-to-event outcome

Other analyses	17	Report other analyses done—eg analyses of subgroups and interactions, and sensitivity analyses	Results, reason-specific analyses, adult sensitivity analyses, and exploratory PI product selection; Supplementary Tables S3–S9 and Figures S5–S11
**Discussion**			
Key results	18	Summarise key results with reference to study objectives	Discussion, opening paragraphs
Limitations	19	Discuss limitations of the study, taking into account sources of potential bias or imprecision. Discuss both direction and magnitude of any potential bias	Discussion, final limitations paragraph
Interpretation	20	Give a cautious overall interpretation of results considering objectives, limitations, multiplicity of analyses, results from similar studies, and other relevant evidence	Discussion; Conclusion
Generalisability	21	Discuss the generalisability (external validity) of the study results	Discussion, macro-region interpretation and limitations; Conclusion
**Other information**			
Funding	22	Give the source of funding and the role of the funders for the present study and, if applicable, for the original study on which the present article is based	Funding section

*Give information separately for exposed and unexposed groups.

Note: An Explanation and Elaboration article discusses each checklist item and gives methodological background and published examples of transparent reporting. The STROBE checklist is best used in conjunction with this article (freely available on the Web sites of PLoS Medicine at http://www.plosmedicine.org/, Annals of Internal Medicine at http://www.annals.org/, and Epidemiology at http://www.epidem.com/). Information on the STROBE Initiative is available at www.strobe-statement.org.
Checklist locations refer to sections, tables, figures, or supplementary materials in Manuscript.docx.
