# Supplementary material for: Passive Immunization Hesitancy Among Rabies-Exposed Individuals in China: A Multicenter Cross-Sectional Study
Source: Trop Med Infect Dis. 2026 Aug 24;11(9):238. doi: 10.3390/tropicalmed11090238 (PMC13611066; doi:10.3390/tropicalmed11090238)

Tropical Medicine and Infectious Disease

MDPI

Supplementary Figures

Supplementary Figure S1. Geographic distribution of 585 adult respondents across participating provinces. The map shows the enrolled study sites.

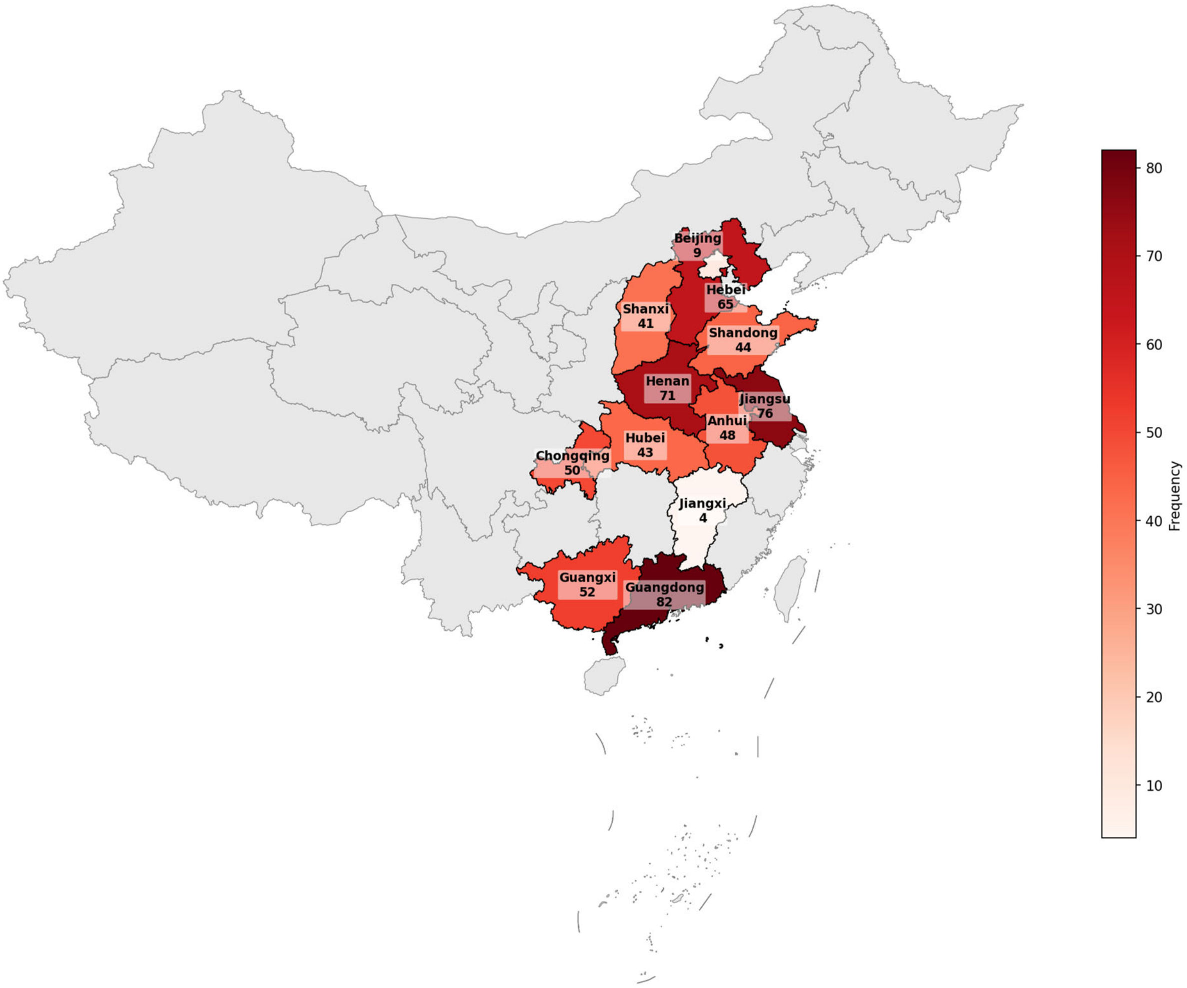

Supplementary Figure S2. Participant flow from eligible Category III exposures to the adult analytic population.

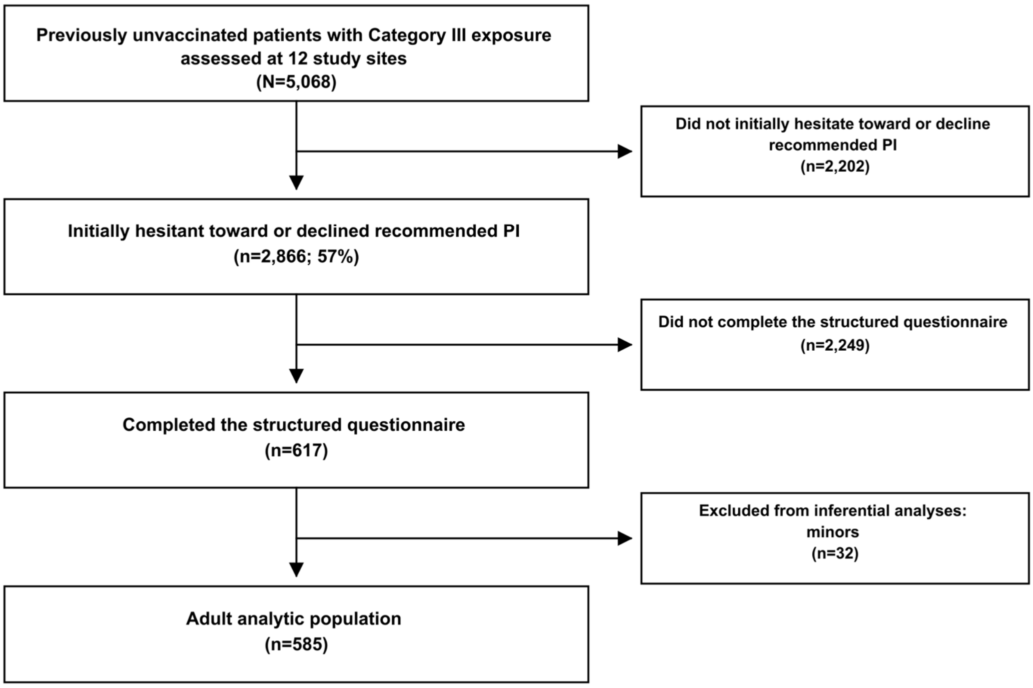

Supplementary Figure S3. Adult respondent count by study site (N=585).

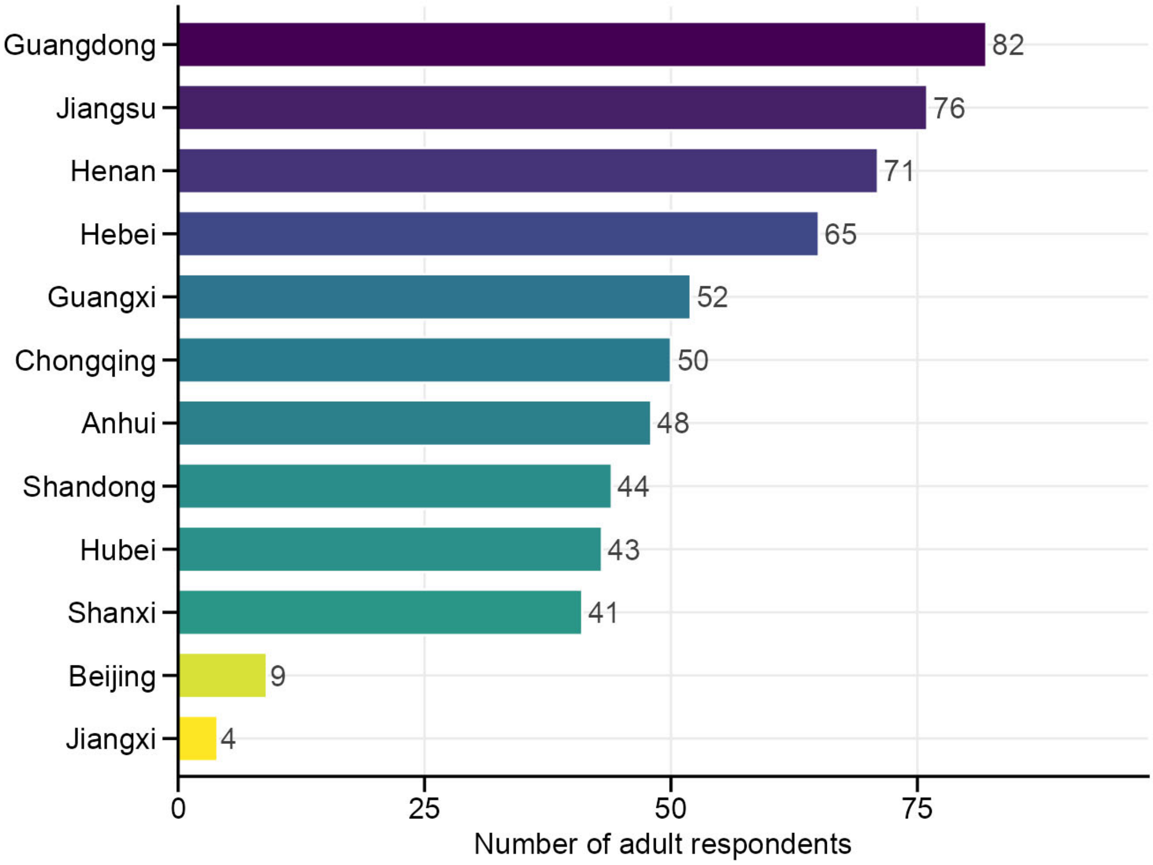

Supplementary Figure S4. Province-specific PI acceptance rates among 585 adults. Points show observed proportions, error bars show Wilson 95% CIs, and point size reflects the province sample size. The dashed line shows the overall adult acceptance rate (320/585, 54.7%).

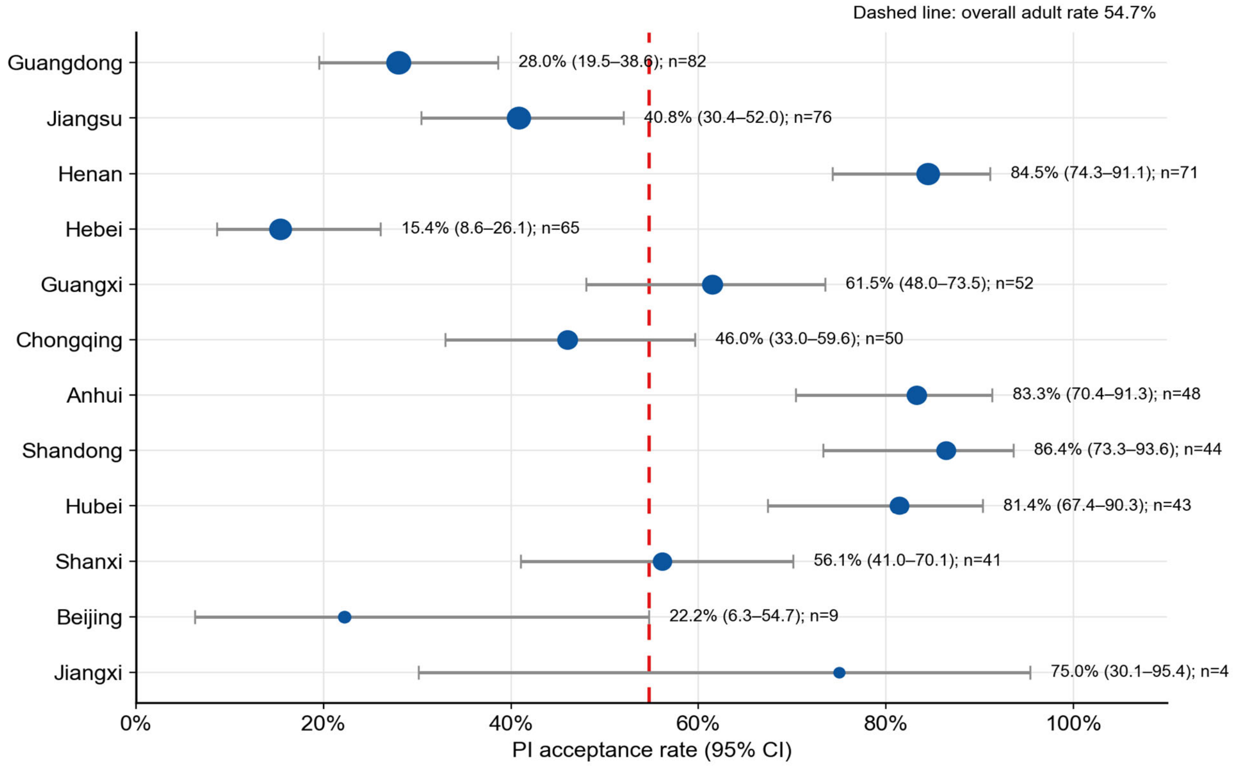

Supplementary Figure S5. Age distribution of 585 adults, stratified by PI treatment decision. The dotted line marks age 18 years.

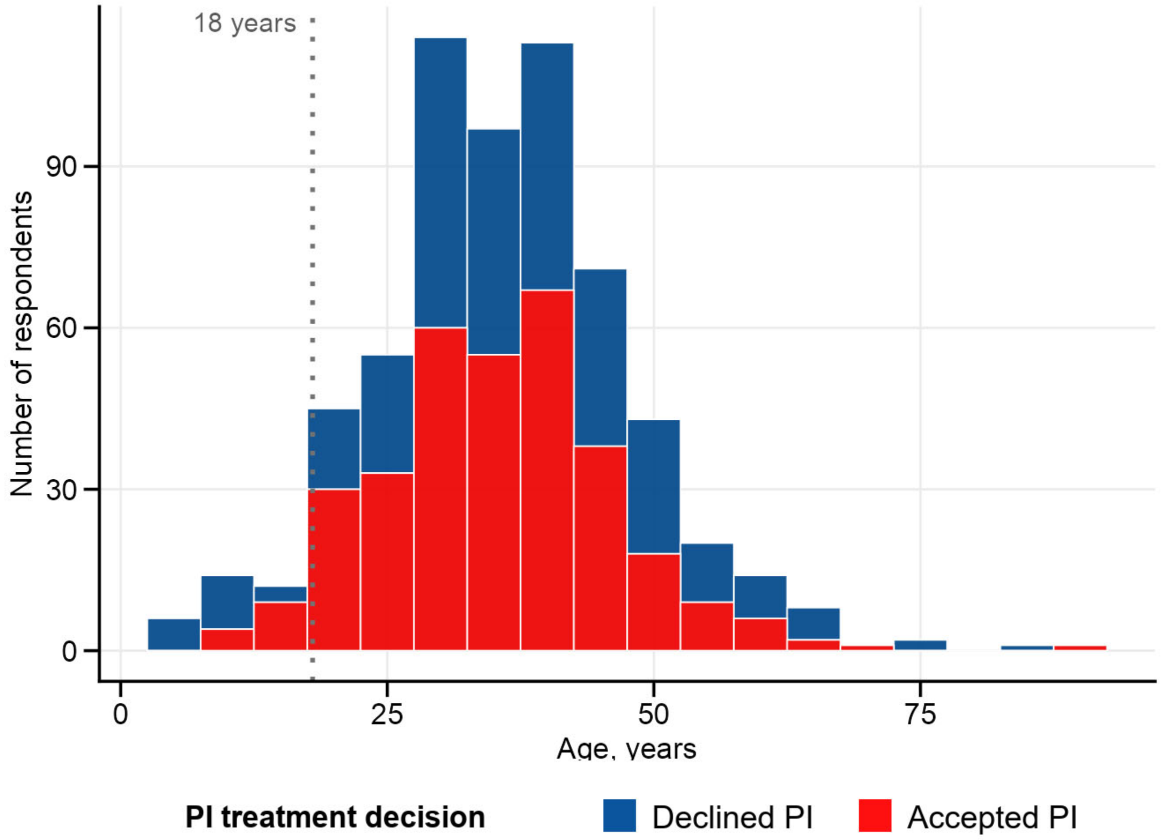

Supplementary Figure S6. Co-occurrence of reported reasons for initial PI hesitancy or refusal among 585 adults. The triangle displays pairwise overlaps among the eight multiple-response reasons.

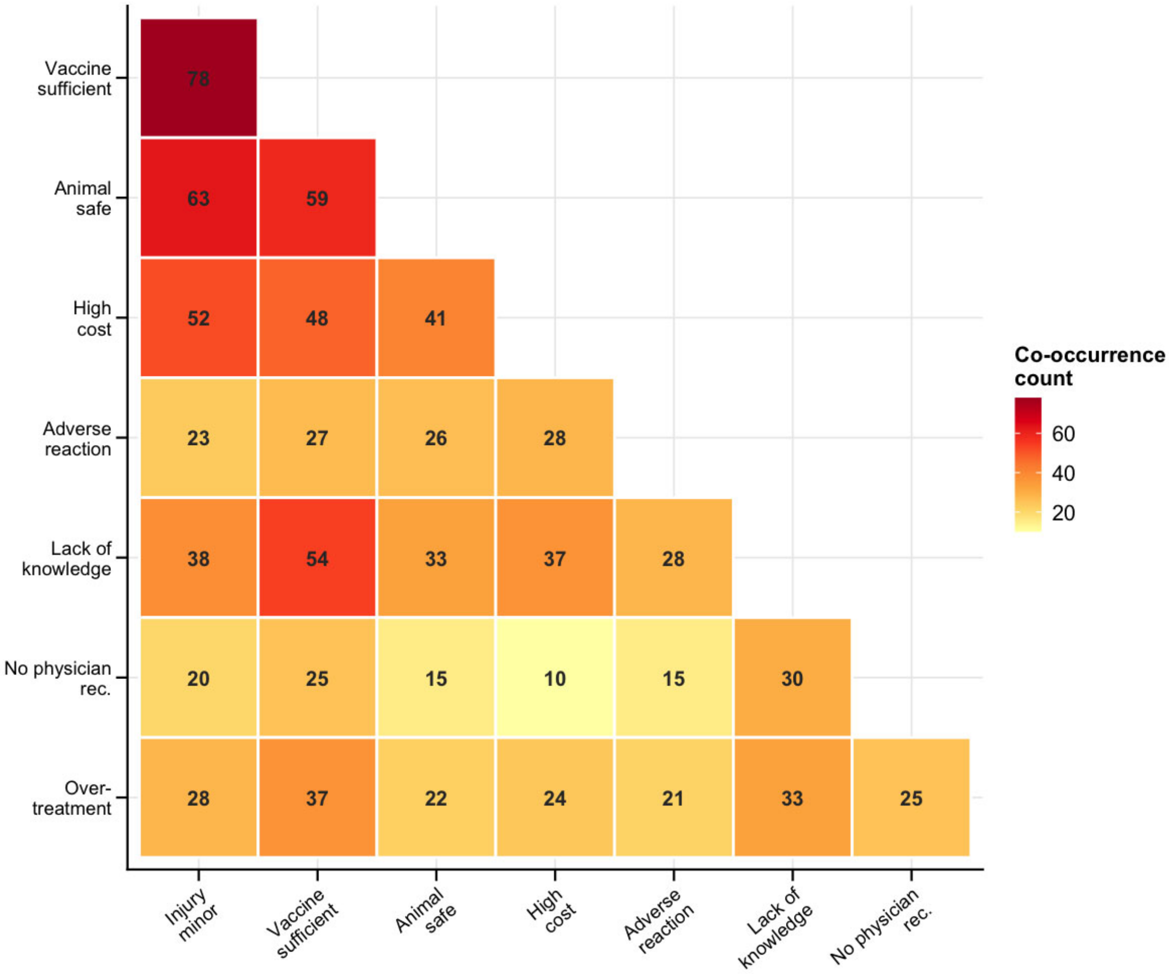

Supplementary Figure S7. Awareness of the early protection gap and PI acceptance among 585 adults. Error bars show Wilson 95% CIs.

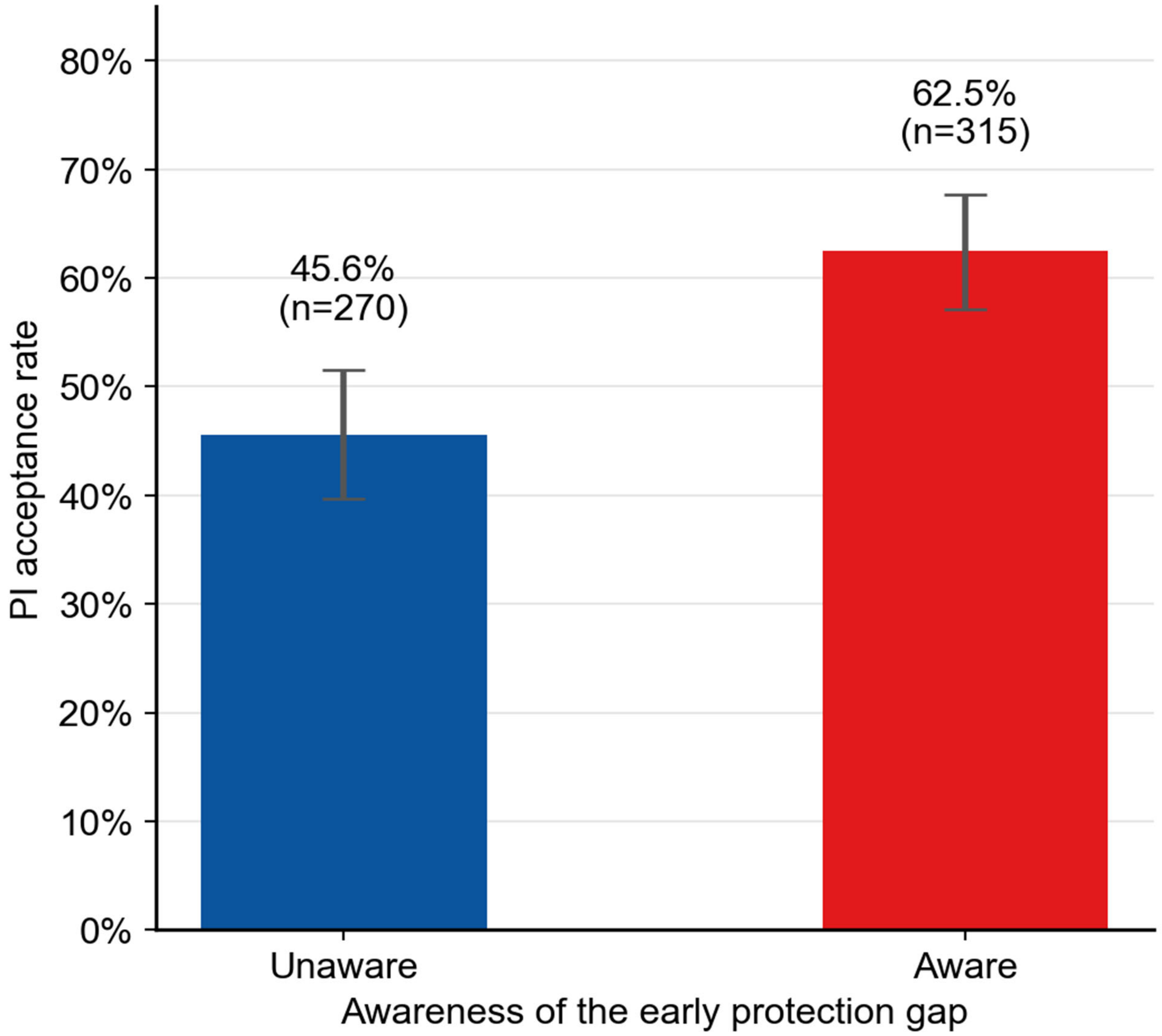

Supplementary Figure S8. Reason-specific Model 2 for the PI treatment decision among 585 adults. The model adjusted for age, monthly household income, macro-region, and awareness of the early protection gap. Open red circles identify reasons that remained significant after BH-FDR correction.

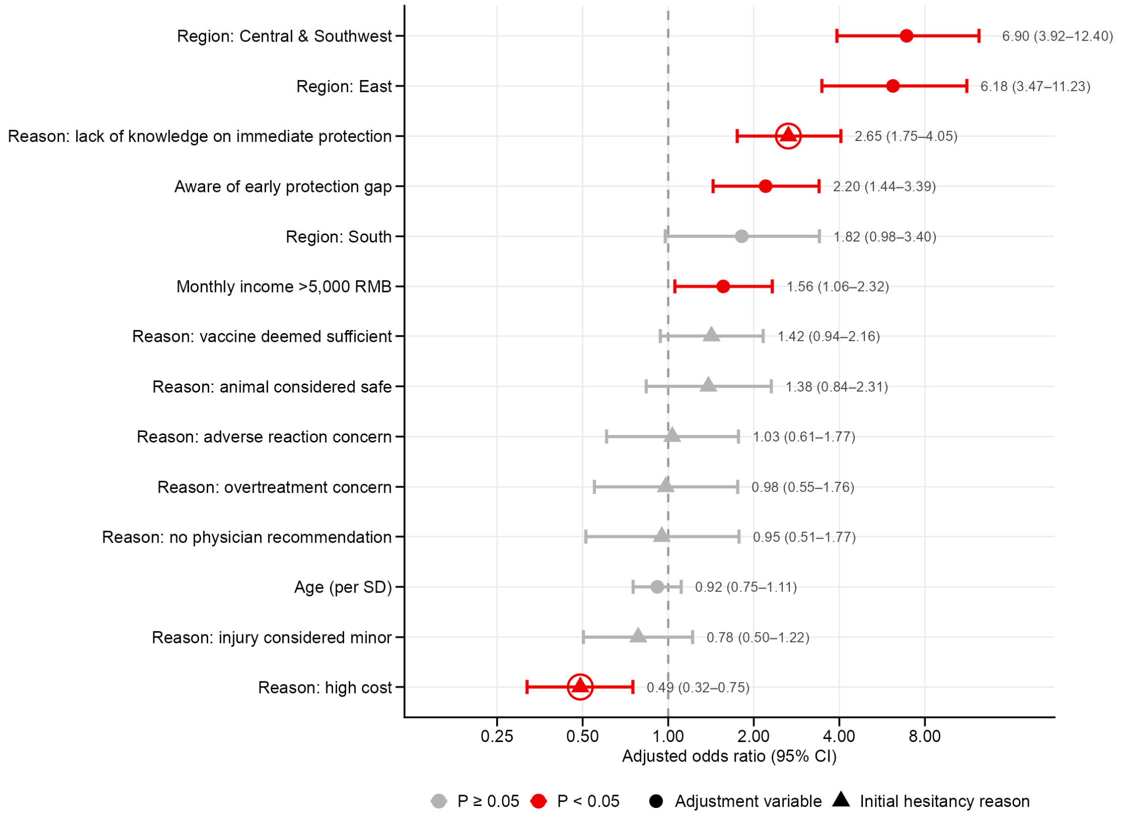

Supplementary Figure S9. PI acceptance rates by preferred future communication channel among adult respondents. Multiple channels could be selected; denominators therefore vary by channel. Error bars show Wilson 95% CIs, point size reflects the channel-specific denominator, and the dashed line shows the overall adult acceptance rate.

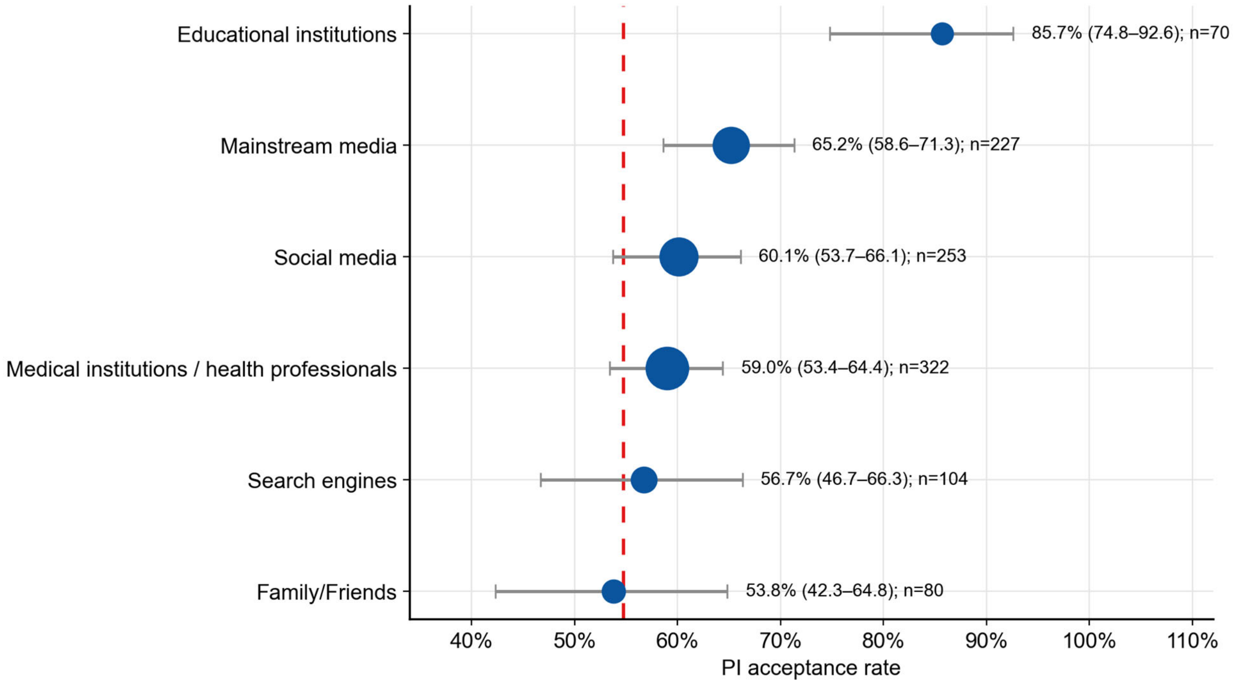

Supplementary Figure S10. PI treatment decision among all 585 adults and PI product selection among the 320 adults who chose PI.

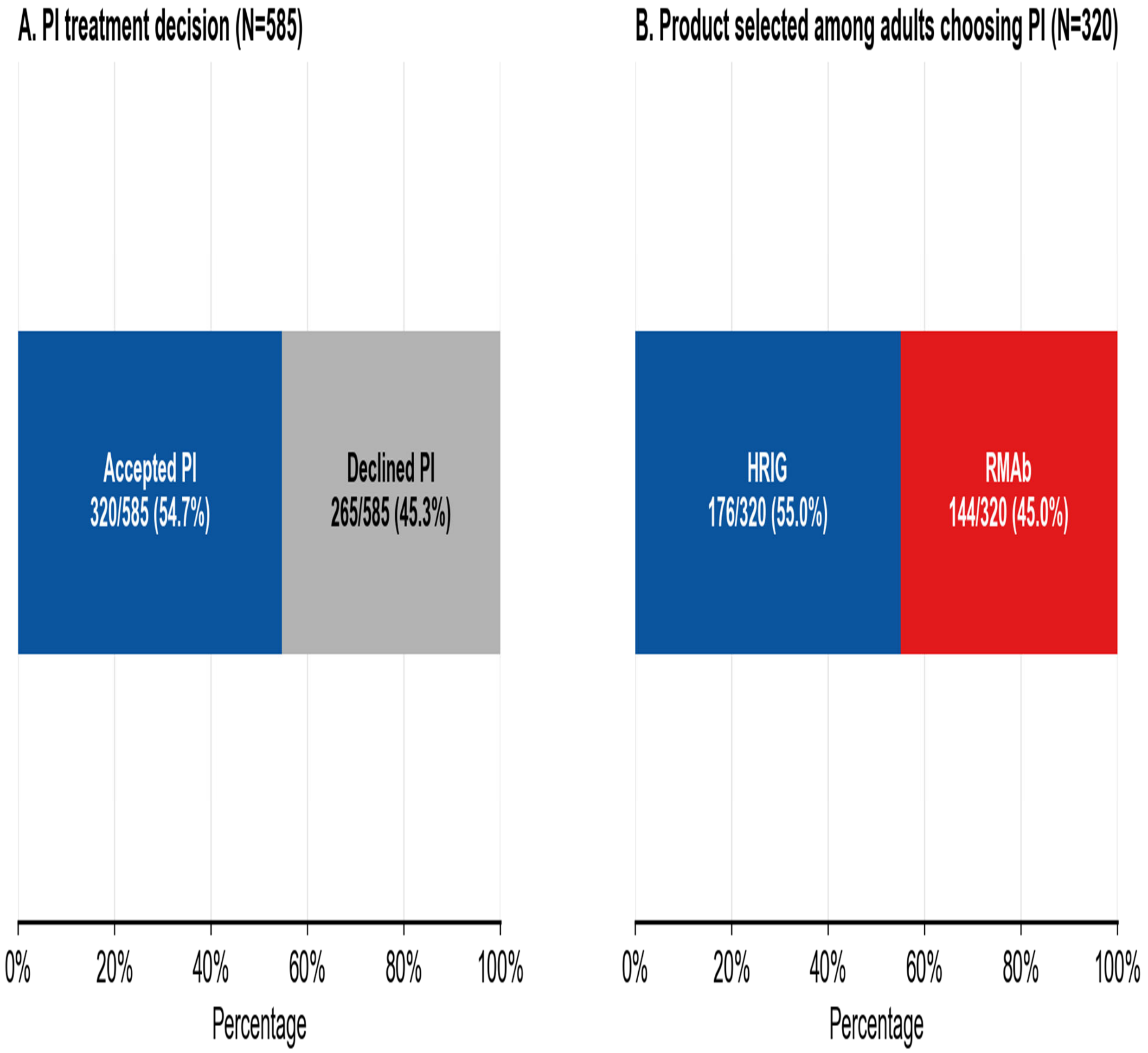

Supplementary Figure S11. HRIG and RMAb selection by monthly household income among the 320 adults who chose PI.

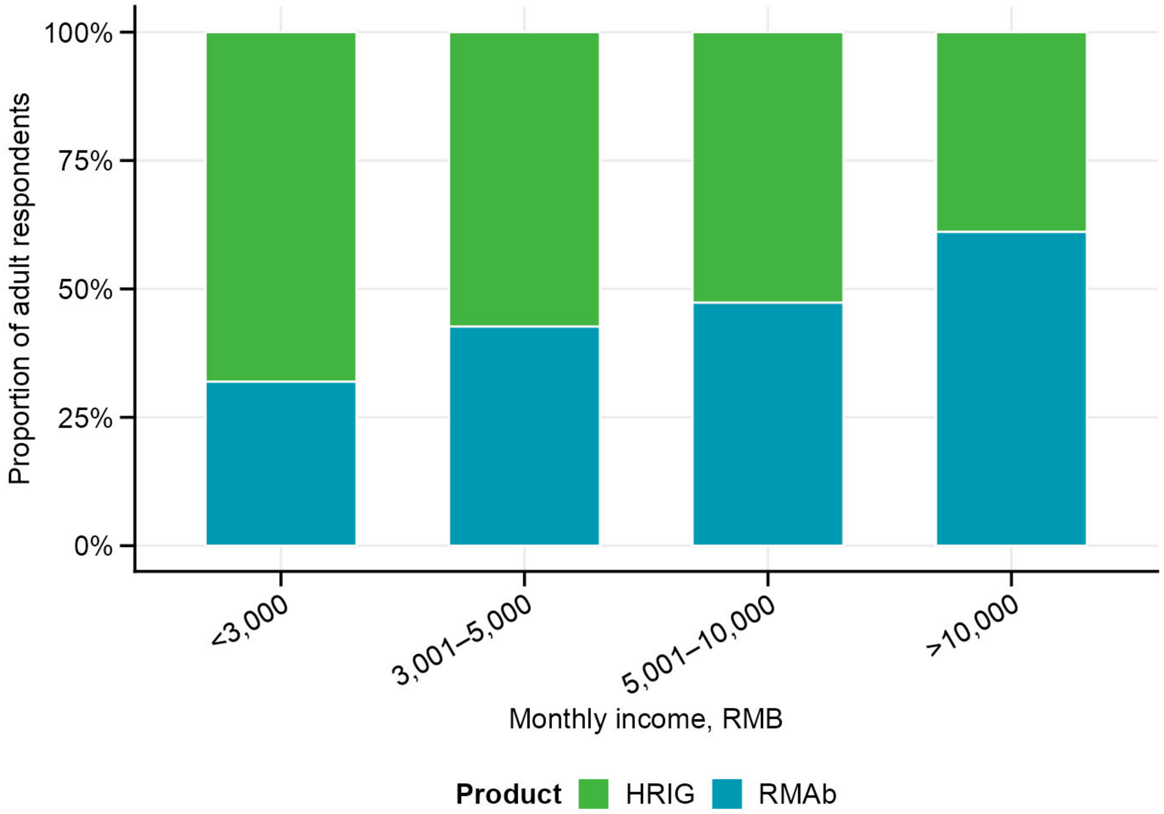

Supplementary Figure S12. HRIG and RMAb selection by educational attainment among the 320 adults who chose PI.

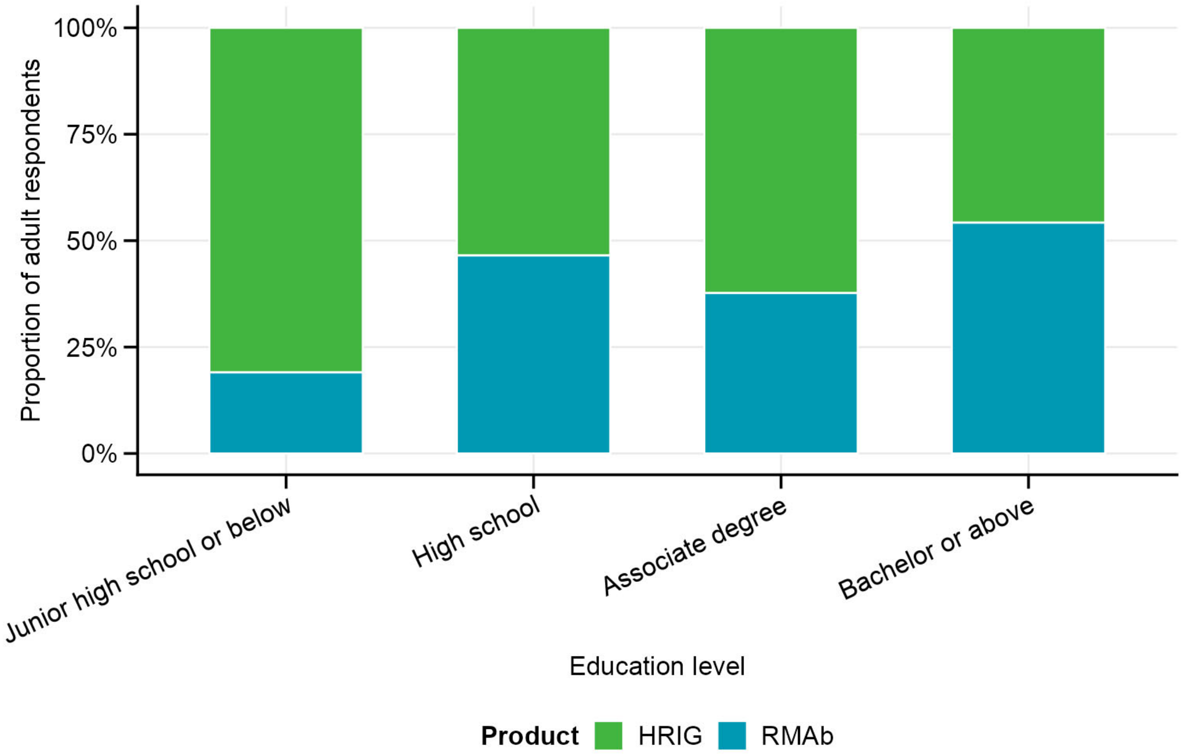

Supplement: Supplementary file 1 [file tropicalmed-11-00238-s001.zip › Supplementary Figures with Legends.pdf]
