# Supplementary material for: Passive Immunization Hesitancy Among Rabies-Exposed Individuals in China: A Multicenter Cross-Sectional Study
Source: Trop Med Infect Dis. 2026 Aug 24;11(9):238. doi: 10.3390/tropicalmed11090238 (PMC13611066; doi:10.3390/tropicalmed11090238)
Supplement: Supplementary file 1 [file tropicalmed-11-00238-s001.zip › Supplementary Material S1 Questionnaire.pdf]

Supplementary Material S1. Questionnaire for patients refusing passive immunization

| Section | Question | Options |
|---|---|---|
| Geographic Information | *Q1: What province is the medical institution where you sought treatment located? | ○ Beijing<br>○ Hebei<br>○ Shandong<br>○ Guangdong<br>○ Hubei<br>○ Jiangxi<br>○ Henan<br>○ Guangxi<br>○ Chongqing<br>○ Shanxi<br>○ Anhui<br>○ Jiangsu |
| Basic Information | *Q2: Gender | ○ Male<br>○ Female |
| | *Q3: Age | ○ _____ (fill in) |
| | *Q4: Please select your current occupational background | ○ Student/Child<br>○ Worker<br>○ Farmer<br>○ Intellectual Worker<br>○ Self-Employed<br>○ Unemployed/Retired<br>○ Other |
| | *Q5: Highest education level | ○ Doctorate and Postdoctoral<br>○ Master<br>○ Bachelor<br>○ Associate Degree<br>○ High School<br>○ Junior High School<br>○ Primary School<br>○ Other |
| | *Q6: Monthly pre-tax income | ○ Below 3000 RMB<br>○ 3001-5000 RMB<br>○ 5001-10000 RMB<br>○ 10001-20000 RMB<br>○ Above 20000 RMB |
| Injury Assessment | Q7: What type of injury severity do you have? | ○ 1 Wound<br>○ 2-3 Wounds<br>○ More than 3 Wounds |
| | Q8: What is the condition of your wound? | ○ Head_Face<br>○ Special_Sites (fingers/toes/nose tip/ears/genitals)<br>○ Mucosa |

| | | |
|---|---|---|
| | | ○ Extremities.Upper_Limb<br>○ Extremities.Lower_Limb<br>○ Extremities.Digits<br>○ Trunk.Chest<br>○ Other |
| | Q9: What animal caused your injury? | ○ Dog<br>○ Cat<br>○ Other |
| | Q10: What type of animal caused your injury? | ○ Owned<br>○ Other Owned<br>○ Stray |
| | Q11: What is the vaccination status of the animal that injured you? | ○ Vaccinated<br>○ Not Vaccinated<br>○ Unknown |
| Health Status | Q12: Regarding your health condition | ○ Chronic Diseases (hypertension, diabetes, etc.)<br>○ HIV Infection<br>○ Organ Transplant<br>○ Immunosuppressive Therapy<br>○ Severe Infection/Advanced Liver Disease<br>○ Cancer Treatment (radiotherapy/chemotherapy)<br>○ Long-term Hormone Use<br>○ Pregnancy Planning/Pregnant<br>○ None of the Above |
| Knowledge Assessment | *Q13: Are you aware that rabies vaccination takes 7–10 days to confer protective immunity, during which there is a window of no immune protection that must be bridged by passive immunization to provide immediate protection? | ○ Know<br>○ Don't Know<br>○ Unclear |
| Hesitancy Reasons | *Q14: What are the reasons you did not choose to receive passive immunization? (Multiple choice) | ○ Vaccine is sufficient<br>○ lack of understanding about immediate neutralizing protection from passive immunization<br>○ Passive immunization is expensive<br>○ Injury not serious enough<br>○ Animal is considered safe<br>○ Concerns about adverse reactions<br>○ Concerns about unnecessary treatment |

| | | |
|---|---|---|
| | | ○ Doctor did not recommend it |
| Information Sources | *Q15: Through which channels have you previously obtained knowledge about rabies prevention? (Multiple choice) | ○ Medical Institutions or Healthcare Professionals<br>○ Social Media<br>○ Mainstream Media<br>○ Family and Friends<br>○ Search Engines<br>○ Educational Institutions<br>○ Did not know before |
| | *Q16: Which channel is most effective for you to learn about rabies prevention? (Multiple choice) | ○ Medical Institutions or Healthcare Workers<br>○ Social Media<br>○ Mainstream Media<br>○ Search Engines<br>○ Friends and Family<br>○ Schools and Educational Institutions |
| Decision Change | *Q17: After completing this questionnaire, have you changed your mind about using rabies passive immunization? | ○ Still Unwilling<br>○ Changed Mind, Agree to Use |
| | *Q18: If you changed your mind to choose passive immunization, which would you prefer? | ○ Rabies Immunoglobulin (blood product, local virus neutralization, moderate price)<br>○ Rabies Cocktail Monoclonal Antibody (non-blood product, fast-acting, local and systemic virus neutralization, low swelling/pain rate, higher price) |

Note: “*” indicates mandatory questions.
