# Supplementary material for: Passive Immunization Hesitancy Among Rabies-Exposed Individuals in China: A Multicenter Cross-Sectional Study
Source: Trop Med Infect Dis. 2026 Aug 24;11(9):238. doi: 10.3390/tropicalmed11090238 (PMC13611066; doi:10.3390/tropicalmed11090238)
Supplement: Supplementary file 1 [file tropicalmed-11-00238-s001.zip › Supplementary Table S1.pdf]

Supplementary Table S1. Original questionnaire response options and coding used in adult analyses (N=585)

Variable	Original questionnaire response options	Coding for analysis	Reference category	Notes
Macro-region (Q1)	Beijing; Hebei; Shandong; Guangdong; Hubei; Jiangxi; Henan; Guangxi; Chongqing; Shanxi; Anhui; Jiangsu	North; East; Central & Southwest; South	North	North: Beijing, Hebei, Shanxi; East: Anhui, Jiangsu, Jiangxi, Shandong; Central & Southwest: Chongqing, Henan, Hubei; South: Guangdong, Guangxi.
Sex (Q2)	Male; Female	Male; female	Female	Used as reported.
Age (Q3)	Free-text age in years	Continuous age standardized for regression; 1 SD=10.98 years	Per 1-SD increase	Age groups of 18–30, 31–45, 46–60, and >60 years were used for descriptive reporting only.
Occupation (Q4)	Student/Child; Worker; Farmer; Intellectual Worker; Self-Employed; Unemployed/Retired; Other	Manual worker/farmer; professional/self-employed; student; unemployed/retired/other	Manual worker/farmer	Worker and farmer were combined; intellectual worker and self-employed were combined; unemployed/retired and other were combined.
Education (Q5)	Doctorate and Postdoctoral; Master; Bachelor; Associate Degree; High School; Junior High School; Primary School; Other	Bachelor's degree or higher; lower than bachelor's degree	Lower than bachelor's degree	For descriptive reporting, Junior High School, Primary School, and Other (no formal education or illiteracy in the source coding) were combined as junior high school or below. Bachelor, master, and doctorate/postdoctoral were combined for regression.
Monthly household income (Q6)	Below CNY 3,000; CNY 3,001–5,000; CNY 5,001–10,000; CNY 10,001–20,000; above CNY 20,000	≤CNY 5,000; >CNY 5,000 for regression	≤CNY 5,000	The original categories were retained for descriptive reporting, with the two highest categories combined as >CNY 10,000.
Number of wounds (Q7)	1 wound; 2–3 wounds; more than 3 wounds	Ordered score corresponding to the three questionnaire categories	Per one-category increase	The original categories were retained for descriptive reporting.
Head/face wound (Q8)	Head_Face; Special_Sites; Mucosa; Extremities.Upper_Limb; Extremities.Lower_Limb; Extremities.Digits; Trunk.Chest; Other	Head/face wound present; absent	Absent	Head_Face was coded as present; all other selected sites were coded as absent for this binary variable.
Animal species (Q9)	Dog; Cat; Other	Dog; cat; other	Not applicable	Used for descriptive comparisons; not included in the prespecified full model.
Animal ownership (Q10)	Owned; Other Owned; Stray	Owned; stray	Owned	Owned and Other Owned were combined.
Animal vaccination status (Q11)	Vaccinated; Not Vaccinated; Unknown	Vaccinated; not vaccinated; unknown	Vaccinated	Unknown was retained as an explicit response category.
Comorbidity or special health condition (Q12)	Chronic Diseases; HIV Infection; Organ Transplant; Immunosuppressive Therapy; Severe Infection/Advanced Liver Disease; Cancer Treatment; Long-term Hormone Use; Pregnancy Planning/Pregnant; None of the Above	Present; absent	Absent	Present when any listed condition was selected; absent when None of the Above was selected.
Awareness of the early protection gap (Q13)	Know; Don't Know; Unclear	Aware; unaware	Unaware	Know was coded as aware; Don't Know and Unclear were combined as unaware.
Initial PI hesitancy reasons (Q14)	Vaccine is sufficient; lack of understanding about immediate neutralizing protection from passive immunization; passive immunization is expensive; injury not serious enough; animal is considered safe; concerns about adverse reactions; concerns about unnecessary treatment; doctor did not recommend it	Each reason coded as selected; not selected	Not selected	Multiple responses were allowed; each reason was analyzed as a separate binary variable.
Previous knowledge sources (Q15)	Medical Institutions or Healthcare Professionals; Social Media; Mainstream Media; Family and Friends; Search Engines; Educational Institutions; Did not	Each source coded as selected; not selected	Not selected	Multiple responses were allowed; 'Did not know before' was retained as a distinct recorded response.

	know before			
Preferred communication channels (Q16)	Medical Institutions or Healthcare Workers; Social Media; Mainstream Media; Search Engines; Friends and Family; Schools and Educational Institutions	Each channel coded as selected; not selected	Not selected	Multiple responses were allowed.
PI treatment decision (Q17)	Still Unwilling; Changed Mind, Agree to Use	Declined PI; chose PI during the same PEP encounter	Declined PI	Outcome for PI treatment-decision analyses.
PI product selection (Q18)	Rabies Immunoglobulin (blood product, local virus neutralization, moderate price); Rabies Cocktail Monoclonal Antibody (non-blood product, fast-acting, local and systemic virus neutralization, low swelling/pain rate, higher price)	HRIG; RMAb among the 320 adults who chose PI	HRIG	Conditional analysis among adults who chose PI.

Note: For every analyzed variable, ‘unknown’ denotes an explicit response that the information was not known, while missing denotes the absence of a recorded response or value. The adult analytic dataset contained no missing values for the variables included in the analyses. AIC, Akaike information criterion; HRIG, human rabies immunoglobulin; PI, passive immunization; RMAb, rabies monoclonal antibody.
