# Supplementary material for: Passive Immunization Hesitancy Among Rabies-Exposed Individuals in China: A Multicenter Cross-Sectional Study
Source: Trop Med Infect Dis. 2026 Aug 24;11(9):238. doi: 10.3390/tropicalmed11090238 (PMC13611066; doi:10.3390/tropicalmed11090238)
Supplement: Supplementary file 1 [file tropicalmed-11-00238-s001.zip › Supplementary Table S2.pdf]

Supplementary Table S2. Prespecified full multivariable logistic regression model for the PI treatment decision among adults (N=585)

Variable	aOR	95% CI	*P* value
Male sex	0.85	0.58–1.24	0.393
Age (per SD)	0.98	0.78–1.21	0.822
**Bachelor's degree or above**	**1.61**	**1.07–2.43**	**0.023**
Monthly income >5,000 RMB	1.45	0.94–2.22	0.090
Professional/self-employed	0.78	0.50–1.22	0.281
Student	1.56	0.66–3.76	0.315
Unemployed/retired/other	1.05	0.59–1.89	0.859
Number of wounds	1.22	0.91–1.64	0.192
**Head/face wound**	**0.53**	**0.30–0.92**	**0.026**
Stray animal	1.28	0.66–2.48	0.471
Animal vaccination: not vaccinated	1.20	0.74–1.96	0.471
Animal vaccination: unknown	1.41	0.83–2.41	0.208
Comorbidity or special health condition: present	1.09	0.62–1.95	0.759
**Aware of early protection gap**	**2.63**	**1.75–3.98**	**<0.001**
**Macro-region: East**	**6.86**	**3.81–12.66**	**<0.001**
**Macro-region: Central & Southwest**	**7.22**	**4.08–13.05**	**<0.001**
**Macro-region: South**	**2.50**	**1.35–4.70**	**0.004**
