# Supplementary material for: Passive Immunization Hesitancy Among Rabies-Exposed Individuals in China: A Multicenter Cross-Sectional Study
Source: Trop Med Infect Dis. 2026 Aug 24;11(9):238. doi: 10.3390/tropicalmed11090238 (PMC13611066; doi:10.3390/tropicalmed11090238)
Supplement: Supplementary file 1 [file tropicalmed-11-00238-s001.zip › Supplementary Table S3.pdf]

Supplementary Table S3. AIC-based adult sensitivity model for the PI treatment decision (N=585)

Variable	aOR	95% CI	P value
Bachelor's degree or above	1.65	1.15–2.37	0.007
Number of wounds	1.24	0.93–1.67	0.140
Head/face wound	0.55	0.32–0.95	0.033
Stray animal	1.63	0.93–2.92	0.093
Aware of early protection gap	2.58	1.74–3.85	<0.001
Macro-region: East	6.52	3.75–11.59	<0.001
Macro-region: Central & Southwest	6.81	3.97–11.93	<0.001
Macro-region: South	2.50	1.39–4.55	0.002
