# Supplementary material for: Passive Immunization Hesitancy Among Rabies-Exposed Individuals in China: A Multicenter Cross-Sectional Study
Source: Trop Med Infect Dis. 2026 Aug 24;11(9):238. doi: 10.3390/tropicalmed11090238 (PMC13611066; doi:10.3390/tropicalmed11090238)
Supplement: Supplementary file 1 [file tropicalmed-11-00238-s001.zip › Supplementary Table S4.pdf]

Supplementary Table S4. Prespecified adult model for the PI treatment decision with province-clustered robust standard errors (N=585)

Variable	Log-odds estimate	Cluster-robust SE	aOR	Robust 95% CI	P value
Male sex	-0.168	0.167	0.85	0.61–1.17	0.315
Age (per SD)	-0.025	0.074	0.98	0.84–1.13	0.736
Bachelor's degree or above	0.476	0.277	1.61	0.94–2.77	0.085
Monthly income >5,000 RMB	0.369	0.222	1.45	0.94–2.23	0.096
Professional/self-employed	-0.248	0.184	0.78	0.54–1.12	0.177
Student	0.445	0.695	1.56	0.40–6.09	0.522
Unemployed/retired/other	0.053	0.346	1.05	0.53–2.08	0.879
Number of wounds	0.197	0.189	1.22	0.84–1.76	0.297
Head/face wound	-0.644	0.140	0.53	0.40–0.69	<0.001
Stray animal	0.243	0.564	1.28	0.42–3.85	0.667
Animal vaccination: not vaccinated	0.180	0.202	1.20	0.81–1.78	0.373
Animal vaccination: unknown	0.342	0.212	1.41	0.93–2.13	0.107
Comorbidity or special health condition: present	0.090	0.300	1.09	0.61–1.97	0.764
Aware of early protection gap	0.967	0.294	2.63	1.48–4.68	0.001
Macro-region: East	1.925	0.671	6.86	1.84–25.54	0.004
Macro-region: Central & Southwest	1.977	0.702	7.22	1.83–28.55	0.005
Macro-region: South	0.916	0.585	2.50	0.79–7.87	0.117

HC0 cluster-robust standard errors clustered by province (k = 12 clusters). Robust 95% CI calculated from cluster-robust SE.
