# Supplementary material for: Passive Immunization Hesitancy Among Rabies-Exposed Individuals in China: A Multicenter Cross-Sectional Study
Source: Trop Med Infect Dis. 2026 Aug 24;11(9):238. doi: 10.3390/tropicalmed11090238 (PMC13611066; doi:10.3390/tropicalmed11090238)
Supplement: Supplementary file 1 [file tropicalmed-11-00238-s001.zip › Supplementary Table S5.pdf]

Supplementary Table S5. Animal vaccination status among adults with and without two reported reasons for initial PI hesitancy or refusal (N=585)

Reported reason	Reason selected: vaccinated animal, n/N (%)	Reason not selected: vaccinated animal, n/N (%)	P value
Animal considered safe	73/114 (64.0%)	248/471 (52.7%)	0.0019
No physician recommendation	23/64 (35.9%)	298/521 (57.2%)	0.0040

P values are unadjusted.
