# Supplementary material for: Passive Immunization Hesitancy Among Rabies-Exposed Individuals in China: A Multicenter Cross-Sectional Study
Source: Trop Med Infect Dis. 2026 Aug 24;11(9):238. doi: 10.3390/tropicalmed11090238 (PMC13611066; doi:10.3390/tropicalmed11090238)
Supplement: Supplementary file 1 [file tropicalmed-11-00238-s001.zip › Supplementary Table S6.pdf]

Supplementary Table S6. Selected concerns by head/face wound status among adults (N=585)

Reported concern	No head/face wound, N=515, n (%)	Head/face wound, N=70, n (%)	P value
Adverse reaction concern	74 (14.4%)	12 (17.1%)	0.539
Overtreatment concern	70 (13.6%)	7 (10.0%)	0.404

P values are unadjusted.
