# Supplementary material for: Passive Immunization Hesitancy Among Rabies-Exposed Individuals in China: A Multicenter Cross-Sectional Study
Source: Trop Med Infect Dis. 2026 Aug 24;11(9):238. doi: 10.3390/tropicalmed11090238 (PMC13611066; doi:10.3390/tropicalmed11090238)
Supplement: Supplementary file 1 [file tropicalmed-11-00238-s001.zip › Supplementary Table S7.pdf]

Supplementary Table S7. Complete reason-specific Model 1 for the PI treatment decision without adjustment for early-protection-gap awareness (N=585)

Variable	aOR	95% CI	P value	BH-FDR P value
**Reason: lack of knowledge on immediate protection**	**2.43**	**1.62–3.68**	**<0.001**	**<0.001**
Reason: vaccine deemed sufficient	1.58	1.05–2.38	0.029	0.076
**Reason: high cost**	**0.41**	**0.27–0.62**	**<0.001**	**<0.001**
Reason: injury considered minor	0.82	0.53–1.26	0.361	0.578
Reason: animal considered safe	1.45	0.88–2.41	0.146	0.291
Reason: adverse reaction concern	0.98	0.58–1.67	0.951	0.951
Reason: overtreatment concern	1.02	0.58–1.82	0.945	0.951
Reason: no physician recommendation	0.95	0.52–1.75	0.861	0.951
Age (per SD)	0.89	0.73–1.07	0.214	—
Monthly income >5,000 RMB	1.50	1.02–2.22	0.040	—
Macro-region: East	4.68	2.71–8.25	<0.001	—
Macro-region: Central & Southwest	6.23	3.57–11.11	<0.001	—
Macro-region: South	1.23	0.69–2.19	0.491	—

BH-FDR correction applied to the 8 reason-specific comparisons only; adjustment variables excluded.
