# Supplementary material for: Passive Immunization Hesitancy Among Rabies-Exposed Individuals in China: A Multicenter Cross-Sectional Study
Source: Trop Med Infect Dis. 2026 Aug 24;11(9):238. doi: 10.3390/tropicalmed11090238 (PMC13611066; doi:10.3390/tropicalmed11090238)
Supplement: Supplementary file 1 [file tropicalmed-11-00238-s001.zip › Supplementary Table S8.pdf]

Supplementary Table S8. Complete reason-specific Model 2 for the PI treatment decision with adjustment for early-protection-gap awareness (N=585)

Variable	aOR	95% CI	P value	BH-FDR P value
**Reason: lack of knowledge on immediate protection**	**2.65**	**1.75–4.05**	**<0.001**	**<0.001**
Reason: vaccine deemed sufficient	1.42	0.94–2.16	0.099	0.263
**Reason: high cost**	**0.49**	**0.32–0.75**	**0.001**	**0.004**
Reason: injury considered minor	0.78	0.50–1.22	0.280	0.449
Reason: animal considered safe	1.38	0.84–2.31	0.207	0.414
Reason: adverse reaction concern	1.03	0.61–1.77	0.904	0.947
Reason: overtreatment concern	0.98	0.55–1.76	0.947	0.947
Reason: no physician recommendation	0.95	0.51–1.77	0.869	0.947
Aware of early protection gap	2.20	1.44–3.39	<0.001	—
Age (per SD)	0.92	0.75–1.11	0.375	—
Monthly income >5,000 RMB	1.56	1.06–2.32	0.027	—
Macro-region: East	6.18	3.47–11.23	<0.001	—
Macro-region: Central & Southwest	6.90	3.92–12.40	<0.001	—
Macro-region: South	1.82	0.98–3.40	0.061	—

BH-FDR correction applied to the 8 reason-specific comparisons only; adjustment variables excluded.
