# Supplementary material for: Passive Immunization Hesitancy Among Rabies-Exposed Individuals in China: A Multicenter Cross-Sectional Study
Source: Trop Med Infect Dis. 2026 Aug 24;11(9):238. doi: 10.3390/tropicalmed11090238 (PMC13611066; doi:10.3390/tropicalmed11090238)
Supplement: Supplementary file 1 [file tropicalmed-11-00238-s001.zip › Supplementary Table S9.pdf]

Supplementary Table S9. Exploratory multivariable model of RMAb versus HRIG selection among adults who chose PI (N=320)

Variable	aOR	95% CI	P value
Male sex	1.18	0.72–1.97	0.509
Age (per SD)	0.92	0.69–1.21	0.545
Bachelor's degree or above	2.11	1.23–3.65	0.007
Monthly income >5,000 RMB	1.31	0.77–2.24	0.322
Head/face wound	0.51	0.22–1.16	0.116
Stray animal	0.64	0.27–1.47	0.295
Animal vaccination: not vaccinated	0.85	0.43–1.69	0.652
Animal vaccination: unknown	1.61	0.80–3.32	0.186
Comorbidity or special health condition: present	2.21	1.04–4.85	0.042
Macro-region: East	13.62	3.68–88.98	<0.001
Macro-region: Central & Southwest	21.14	5.71–138.22	<0.001
Macro-region: South	9.27	2.30–63.18	0.006
